# Analysis of the influence of the COVID-19 pandemic on maternal mortality trends, 2011–2021*

**DOI:** 10.1590/1518-8345.7559.4668

**Published:** 2025-11-03

**Authors:** Mariana Vitor Peppe, Fernanda Bruzadelli Paulino da Costa, Livia Sanches Pedrilio, Caio Antonio de Campos Prado, Ana Cyntia Paulin Baraldi, Juliana Stefanello

**Affiliations:** 1 Universidade de São Paulo, Escola de Enfermagem de Ribeirão Preto, PAHO/WHO Collaborating Centre for Nursing Research Development, Ribeirão Preto, SP, Brazil; 2 Scholarship holder at the Coordenação de Aperfeiçoamento de Pessoal de Nível Superior (CAPES), Brazil; 3 Sociedade Beneficente Israelita Brasileira Albert Einstein, Escritório de Excelência, São Paulo, SP, Brazil; 4 Universidade de São Paulo, Faculdade de Medicina de Ribeirão Preto, Ribeirão Preto, SP, Brazil; 5 Fundação de Apoio ao Ensino, Pesquisa e Assistência, Centro de Referência da Saúde da Mulher-Mater, Ribeirão Preto, SP, Brazil; 6 Universidade de São Paulo, Hospital das Clínicas da Faculdade de Medicina de Ribeirão Preto, Ribeirão Preto, SP, Brazil; 7 Organização Pan-Americana da Saúde, Bireme, Brasília, DF, Brazil

**Keywords:** Maternal Death, COVID-19, Maternal Health, Obstetrics, Epidemiologic Methods, Health Surveillance System

## Abstract

to analyze the time-series trend for maternal mortality before and during the COVID-19 pandemic.

ecological time-series study in the state of São Paulo and in its seventeen Regional Health Departments. Collection of secondary data provided by the Ministry of Health. Study variables: total, direct and indirect maternal mortality ratios. Analysis was performed by segmented regression model.

maternal mortality ratio trends were stationary in 2011–2019, followed by a 25.6% increase in 2019–2021. The direct maternal mortality ratio had an upward trend of 7.2% in 2011–2017 and a stationary trend in 2017–2021. The indirect maternal mortality ratio was stationary in 2011–2019, followed by a 70.5% increase in 2019–2021. The regional health departments of the Metropolitan Area of São Paulo, Araçatuba, Bauru, Campinas, Presidente Prudente, São João da Boa Vista, São José do Rio Preto and Sorocaba showed an upward trend for maternal mortality in 2011–2021.

the COVID-19 pandemic severely influenced the increase in maternal mortality, especially from indirect causes, with 2021 being the worst year in the time series. The findings were important to assist in the organization of maternal mortality reduction strategies by directing them to priority areas.

## Highlights

**(1)** Contribution to the organization of strategies to reduce maternal death. **(2)** Important results for planning and implementing regional initiatives in nursing. **(3)** Direction of priority actions to areas with the worst mortality rates. **(4)** The COVID-19 pandemic severely impacted the increase in maternal death.

## Introduction

In early 2020, the World Health Organization (WHO) announced the detection of a group of pneumonia cases in the city of Wuhan, capital of Hubei Province, China. These cases were associated with a new coronavirus, previously unknown in humans^(1)^. The disease quickly spread to several countries, becoming a significant cause of respiratory infections, pneumonia and deaths. In March 2020, the WHO declared the COVID-19 pandemic^(2-3)^, maintaining the Public Health Emergency of International Concern status until May 2023^(4)^.

The COVID-19 pandemic resulted in a significant increase in the number of deaths from different causes. According to WHO estimates, there were approximately 14.8 million excess deaths. Brazil ranked fifth among the countries with the highest absolute number of deaths beyond expectation and ranked twentieth among the countries with the highest ratio of excess deaths in relation to the expected total^(5)^. This situation directly impacted access to health care systems and its overload, resulting in reduced availability of qualified health care professionals and health resources^(6)^. In the initial period of the pandemic, specialists were focused on determining whether pregnancy could represent a risk factor for serious COVID-19 complications. This concern was based on evidence of similar outcomes observed in infections caused by other respiratory viruses, such as H1N1^(7)^. With the evolution of the pandemic and related research, this theory was confirmed and there was a significant increase in maternal mortality rates^(8-9)^. The increase observed in Brazil probably reflects chronic structural problems that affect women’s health care at different levels of health care services. Among these challenges, we note the difficulty of access to health care services and the low quality of prenatal care^(10)^.

The journey of Brazilian women and health care professionals in the area of obstetrics has been long in seeking safer care for delivery and birth care. The last three decades saw a strengthening of national policies aimed at maternal and child health, focusing on expanding access to prenatal care and reproductive planning, aiming to improve the quality of obstetric care and reduce maternal mortality. Among these initiatives, we note the establishment of maternal mortality committees and maternal death surveillance systems, the Prenatal Care and Birth Humanization Program, the National Policy for Comprehensive Care for Women’s Health, the Stork Network^(11)^, and, more recently, the Alyne Network^(12)^. The implementation of these measures contributed to a significant reduction in the Maternal Mortality Ratio (MMR) in Brazil, which declined from 72.4 to 57.9 maternal deaths per 100,000 live births between 2009 and 2019^(13)^.

Reducing maternal mortality rates is a priority at the global level and is part of the 2030 Agenda of the United Nations Sustainable Development Goals (SDGs). The established goal seeks to reduce global maternal mortality ratio to less than 70 deaths per 100,000 live births, serving as a reference for the formulation of national policies and international cooperation initiatives. In 2015, Brazil reaffirmed its commitment to this cause and adjusted the national target, aiming to reduce the Maternal Mortality Ratio to a maximum of 30 deaths per 100,000 live births^(14-15)^. In the years prior to the pandemic, MMR stabilized at levels ranging from 62 deaths per 100,000 live births in 2015 to 57.9 deaths per 100,000 live births in 2019, with small fluctuations from 2011^(16)^. These data raise a red flag about the possibility of Brazil not meeting the self-established goal in relation to the SDGs^(13)^.

Maternal death is defined as the death of women during pregnancy, abortion, childbirth or up to 42 days after the end of pregnancy, regardless of its duration. The cause is associated with conditions related to or aggravated by pregnancy or its management, excluding accidental or incidental factors^(17-18)^. Several factors influence maternal mortality, including access to health care services, the quality of care provided in prenatal care, during labor, childbirth, the puerperium and in obstetric emergencies. In addition, clinical and socioeconomic factors and the incidence of diseases also play a significant role in this context^(19-20)^.

A public health emergency can aggravate the existing situation, raising the risk of pregnancy complications, burdening the health care system and limiting access to maternal and perinatal care^(20-21)^. In this context, the COVID-19 pandemic demanded health care, human, institutional, social, economic and political resources, competing with other demands of the health care system. As a result, maternal mortality was at an even greater risk of being neglected, especially for being strongly related to social inequalities.

Considering the consequences of the COVID-19 pandemic on maternal health, we identified the need to carry out this study to clarify the influence of the pandemic on women’s health during the pregnancy-puerperal cycle in the state of São Paulo. This need, in addition to the lack of regional studies that analyze maternal mortality in the context of the COVID-19 pandemic, justifies this study. The objective of the study was to analyze the time-series trend of maternal mortality before and during the COVID-19 pandemic.

## Method

### Study design

For this study, we adopted an epidemiological approach with an analytical longitudinal ecological study using time series.

### Study location and period

The study had as its unit of analysis the seventeen Regional Health Departments (RHD) of the state of São Paulo, whose territorial area is 248,219.481 km², with 645 municipalities and a population of 44,411,238 people. This state was chosen because it is the most populous state in Brazil, with a Human Development Index (HDI) of 0.806, which is considered very high^(22)^, and has an organized administrative health care network, contemplating a satisfactory health care design in the context of the line of health care for pregnant and puerperal women. The period studied included data from years before the pandemic (2011 to 2019) and during the pandemic (2020 to 2021). For each year, wase considered the interval from January 1 to December 31.

### Population

We collected grouped data for pregnant women, aged between 10 and 49 years, who evolved to childbirth and whose children were live births (LB), regardless of the delivery method, notified in the Live Birth Information System (SINASC). We also collected data for women, in the same age group, who had been pregnant and evolved to maternal death due to pregnancy, delivery and postpartum complications, and were registered in the Mortality Information System (SIM).

### Study variables

Aggregate variables were characterized as: total maternal mortality ratio (TMMR = total number of maternal deaths/total number of LB x 100,000), direct maternal mortality ratio (DMMR = total number of maternal deaths from direct cause/total number of LB x 100,000) and indirect maternal mortality ratio (IMMR = total number of maternal deaths from indirect cause/total number of LB x 100,000). For all selections in SINASC and SIM, the following information was considered: municipality of residence and age group from 10 to 49 years.

The characterization of maternal death was based on the diagnoses established in the 10th Revision of the International Classification of Diseases (ICD-10), codified in chapter XV – Complications of pregnancy, delivery and puerperium, being classified as direct and indirect obstetric death^(18)^. The ICD-10 diagnoses related to pregnancy and puerperium included in other chapters were also considered.

### Data collection

The data were obtained from the Live Birth Information System and the Mortality Information System made available by the Department of Informatics of the Unified Health System (DATASUS), of the Ministry of Health, through the TABNET platform. In SINASC, we collected grouped information for women who had been pregnant, who evolved to childbirth and whose children were LB, regardless of the delivery method. In the SIM, we collected data for women who had been pregnant and who, due to pregnancy, delivery and postpartum complications, evolved to death.

Data collection was conducted between September and November 2023, and was carried out by the researcher herself. During collection, the raw data from the databases were organized and categorized in a Microsoft Office Excel® spreadsheet and subsequently imported for analysis.

### Data analysis

For the time-series data analysis, we adopted segmented regression models (Joinpoint Regression), built to identify points of statistically relevant changes in MMR over time. Models were built considering the dependent variables (maternal mortality ratios) and independent variables (year and RHD). Two analyses were performed, the first considering only the total data for the state of São Paulo, having as dependent variables the maternal mortality ratios (TMMR, DMMR and IMMR) and as independent variable the selected years. A second analysis was performed considering the territorial distribution of maternal mortality ratios, keeping these ratios as dependent variable and RHDs and years as independent variables.

Segmented regression is a statistical modeling technique that seeks to explain the relationship between two variables through regression lines. The points that join these lines are called inflection points^(23)^. This model assumes a linear trend between inflection points and allows adjustment of a weighted regression model. This technique estimates the annual percent change (APC) and the average annual percent change (AAPC)^(24)^.

Confidence intervals of 95% (95% CI) and a significance level of 5% were considered. The model was adjusted so the number of inflection points could range from zero to two, that is, up to three segments. The significance tests for choosing the best model were based on the Monte Carlo permutation method, and the implementation of the models was performed in the Joinpoint Regression Program version 4.3.1.0. The program’s default setting (Grid Search method) automatically selects the number of points needed to adjust each segment^(24)^.

### Ethical aspects

The study was approved by the Research Ethics Committee under Opinion 4,649,272, of April 2021. The data used are aggregated and allow no identification of those involved in the results presented, thus not requiring the application of the Informed Consent Form.

## Results

There were 3,339 maternal deaths in the state of São Paulo, of women in childbearing age range between 10 and 49 years, between 2011 and 2021. Considering the MMR, there was a small variation between 2011 and 2016 (36.65 to 50.70 deaths/100,000 LB), a rise in 2017 (56.72 deaths/100,000 LB), with a decrease by 7 deaths/100,000 LB between 2018 and 2019. A new increase occurred in 2020, by almost 12 deaths/100,000 LB, reaching the highest value in the time series in 2021, with 87.02 deaths/100,000 LB, according to Table 1.

**Table 1-:** Maternal mortality relative frequency and ratios per 100,000 live births between the years. State of São Paulo, SP, Brazil, 2011-2021

	**Years**												
			2011	2012	2013	2014	2015	2016	2017	2018	2019	2020	2021
Absolute numbers:													
Live births*		n	610,150	616,545	610,836	625,590	633,935	652,732	611,735	606,065	583,081	552,210	525,134
Total maternal mortality		n	248	226	240	263	310	331	347	303	282	332	457
Direct maternal death*		n	152	125	146	150	191	199	212	189	158	154	143
		(%)	61.3	55.3	60.8	57.0	61.6	60.1	61.1	62.4	56.0	46.4	31.3
Indirect maternal death*		n	89	97	90	105	113	126	119	104	113	158	301
		(%)	35.9	42.9	37.5	39.9	36.4	38.1	34.3	34.3	40.1	47.6	65.9
Unspecified maternal death*		n	7	4	4	8	6	6	16	10	11	20	13
		(%)	2.8	1.8	1.7	3.0	1.9	1.7	4.6	3.3	3.9	6.0	2.8
													
Total MMR ^†^			40.6	36.6	39.3	42.0	48.9	50.7	56.7	50.0	48.4	60.1	87.0
Direct MMR ^†^			24.9	20.3	23.9	24.0	30.1	30.5	34.6	31.2	27.1	27.9	27.2
Indirect MMR ^†^			14.6	15.7	14.7	16.8	17.8	19.3	19.4	17.1	19.4	28.6	57.3
Unspecified MMR ^†^			1.1	0.6	0.6	1.3	0.94	0.9	2.6	1.6	1.9	3.6	2.5

*Source: DATASUS (2023); ^†^MMR = Maternal Mortality Ratio

**Figure 1-:**
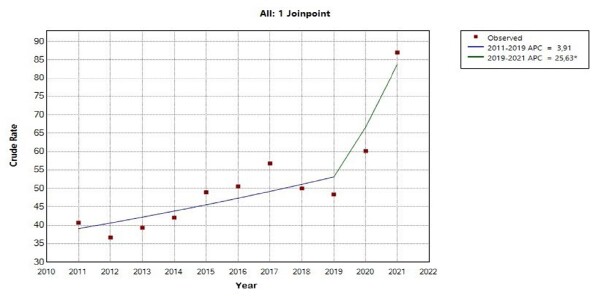
Joinpoint regression model for the total maternal mortality ratio in the state of São Paulo

Figure 1 presents the time-series analysis. The observed results show statistically relevant changes in the trend for total, direct and indirect maternal mortality ratios. TMMR presented an inflection point, dividing the time series into two segments: 2011 to 2019 and 2019 to 2021. The first segment showed a stationary behavior of 3.9% in the annual percent change (95% CI -12.4 – 25.5). The second segment showed an upward trend in TMMR, with a statistically significant increase of 25.6% in the annual percent change (95% CI 4.4 – 43.1), totaling an increase in the average annual percent change of 7.9%, with statistical significance (95% CI 3.9 – 11.4) between 2011 and 2021.

**Figure 2-:**
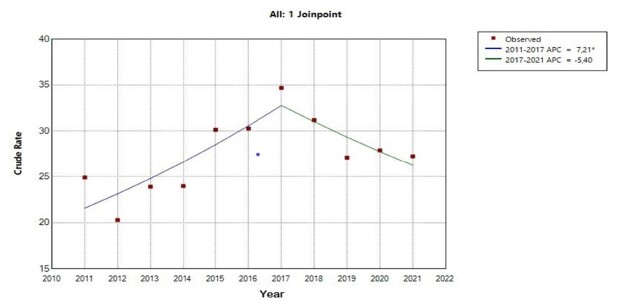
Joinpoint regression model for the direct maternal mortality ratio in the state of São Paulo

Figure 2 shows the representation of the DMMR, in which an inflection point was detected, separating the first segment, corresponding to the years 2011 to 2017, which shows a growth trend with an annual percent change of 7.2% (95% CI 3.9 – 22.8). In the second segment, from 2017 to 2021, although the visual aspect of the curve is downward, statistically the trend was stationary, presenting a percentage of 5.4% in DMMR (95% CI -20.3 – 0.4). The average annual percent change for the entire period remained stationary at 2.0% (95% CI: -1.3 – 5.6).

*Indicates that the Annual Percent Change (APC) is significantly different from zero at the alpha = 0.05 level; Test Statistic and p-value not available for the Empirical Quantile method; final selected model: 1 joinpoint

*Indicates that the Annual Percent Change (APC) is significantly different from zero at the alpha = 0.05 level; Test Statistic and p-value not available for the Empirical Quantile method; final selected model: 1 joinpoint

**Figure 3-:**
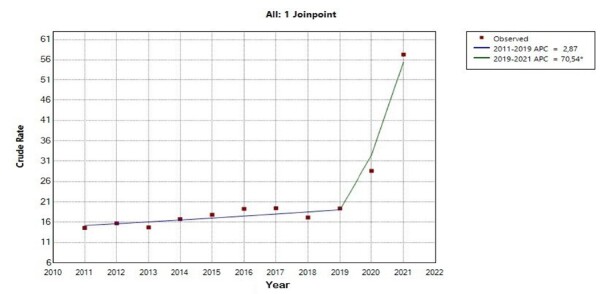
Joinpoint regression model for the indirect maternal mortality ratio in the state of São Paulo

As shown in Figure 3, two segments were identified for IMMR through an inflection point. The first, covering the years 2011 to 2019, presented a stationary behavior in the annual percent change, with a value of 2.9% (95% CI -0.5 – 5.7). The second segment, corresponding to the period from 2019 to 2021, showed a growth trend in the annual percent change of 70.5% (95% CI 51.6 – 87.2). The average annual percent change, in turn, remained in a growth trend, registering 13.8% (95% CI 11.8% - 16.1).

*Indicates that the Annual Percent Change (APC) is significantly different from zero at the alpha = 0.05 level; Test Statistic and p-value not available for the Empirical Quantile method; final selected model: 1 joinpoint

Another segmented regression analysis was performed through the distribution of MMR among the RHDs. Some RHDs did not present maternal death in certain years of the series, thus making it impossible to obtain an individualized model. In the analysis of the distribution of TMMR in RHDs, it was observed that, in RHD 1, there was a trend of annual growth of 5.7% (95% CI 3.7 – 8). In RHD 2, the growth trend was 13.4% (95% CI 5.4 – 24.8). RHDs 6, 11, 14 and 15 also showed a growth trend: 9.5% (95% CI 0.8 - 21.5), 20.01% (95% CI 8.29 – 39.12), 18.8% (95% CI 7.2 - 38.1) and 8% (95% CI 2.2 – 15.6), respectively, with no joinpoints. Although RHDs 3, 4, 9, 13, 17 presented a positive value of APC and AAPC, the trend was considered stationary because there was no statistical significance, as shown in Table 2.

**Table 2-:** Time-series trend for total, direct and indirect maternal mortality ratios, using segmented regression, in the regional health departments. State of São Paulo, SP, Brazil, 2011-2021

**Total maternal mortality ratio**						
**Regional Health Departments**	**Segmented Periods**	**APC** *	**CI** ^†^ **95%**	**AAPC ^‡^ (2011-2021)**	**CI** ^†^ **95%**	Trend APC*/AAPC ^‡^ APC *
						
1 Metropolitan Area of São Paulo	2011–2021	5.7	3.7 ; 8	5.7	3.7 ; 8	Upward/Upward
2 Araçatuba	2011–2021	13.4	5.4 ; 24.8	13.4	5.4 ; 24.8	Upward/Upward
3 Araraquara	2011–2021	2	−10.7 ; 5.9	2	−10.7 ; 15.9	Stationary/Stationary
4 Baixada Santista	2011–2021	7.4	−1.7 ; 18.2	7.4	−1.7 ; 18.2	Stationary/Stationary
6 Bauru	2011–2021	9.5	0.8 ; 21.5	9.5	0.8 ; 21.5	Upward/Upward
7 Campinas	2011–2019	−2.3	−14.4 ; 3.6	8.9	3.4 ; 14.3	Stationary/Upward
	2019–2021	68.7	20.6 ; 18.5			Upward
9 Marília	2011–2021	6.6	−0.2 ; 15.2	6.6	−0.2 ; 15.2	Stationary/Stationary
10 Piracicaba	2011–2013	−36.8	−63.2 ; 6.4	0.6	−7.3 ; 14.5	Stationary/Stationary
	2013–2021	13.1	−25 ; 96.3			Stationary
11 Presidente Prudente	2011–2021	20.0	8.3 ; 9.1	20.0	8.3 ; 9.1	Upward/Upward
13 Ribeirão Preto	2011–2021	9.7	−1.3 ; 25.3	9.7	−1.3 ; 25.3	Stationary/Stationary
14 São João da Boa Vista	2011–2021	18.8	7.2 ; 38.1	18.8	7.2 ; 38.1	Upward/Upward
15 São José do Rio Preto	2011–2021	8.0	2.2 ; 15.6	8.0	2.2 ; 15.6	Upward/Upward
16 Sorocaba	2011–2019	1.8	−39.7 ; 0.5	12.5	3.2 ; 22.8	Stationary/Upward
	2019–2021	68.2	10.3 ; 48.9			Upward
17 Taubaté	2011–2021	7.6	−4.9 ; 23.3	7.6	−4.9 ; 23.3	Stationary/Stationary
						
**Direct maternal mortality ratio**						
1 Metropolitan Area of São Paulo	2011–2017	6.0	2.1 ; 26.2	1.1	−2.6 ; 4.9	Upward/Stationary
	2017–2021	−5.9	−24.1 – 0.5			Stationary
3 Araraquara	2011–2021	−2.9	−19 ; 14.3	−2.9	−19 ; 14.3	Stationary/Stationary
4 Baixada Santista	2011–2021	3.2	−4 ; 11.5	3.2	−4 ; 11.5	Stationary/Stationary
6 Bauru	2011–2019	12.1	−1.9 ; 184.4	−8	−26.9 ; 23.1	Stationary/Stationary
	2019–2021	−58.4	− 88.4 ; 5			Stationary
7 Campinas	2011–2021	1	−5.7 ; 8	1	−5.7 ; 8	Stationary/Stationary
9 Marília	2011–2017	18.9	−4.4 ; 195.5	3.8	−12.2 ; 25.9	Stationary/Stationary
	2017–2021	−15.3	−70.8 ; 16.1			Stationary
10 Piracicaba	2011–2021	1.3	−8 ; 11.7	1.3	−8 ; 11.7	Stationary/Stationary
11 Presidente Prudente	2011–2021	7.2	−10.5 ; 31.3	7.2	−10.5 ; 31.3	Stationary/Stationary
13 Ribeirão Preto	2011–2015	39.8	10.5 ; 343.6	5.3	−11.4 ; 26.2	Upward/Stationary
	2015–2021	−12.9	−53.5 ; −1.8			Downward
15 São José do Rio Preto	2011–2021	2.7	−2.7 ; 8.8	2.7	−2.7 ; 8.8	Stationary/Stationary
16 Sorocaba	2011–2021	7.8	−2.6 ; 22	7.8	−2.6 ; 22	Stationary/Stationary
17 Taubaté	2011–2021	−0.1	−13.2 ; 15.4	−0.1	−13.2 ; 15.4	Stationary/Stationary
						
**Indirect maternal mortality ratio**						
1 Metropolitan Area of São Paulo	2011–2018	2.4	−8.7 ; 7.3	10.5	7.2 ; 13.6	Stationary/Upward
	2018–2021	32.1	16.6 ; 58.8			Upward
4 Baixada Santista	2011–2019	4	−30.3 ; 70	12	3.3 ; 22.5	Stationary/Upward
	2019–2021	50.6	4.4 ; 107.7			Upward
7 Campinas	2011–2019	−4.1	−29.8 ; 8.3	16.4	7.7 ; 27.1	Stationary/Upward
	2019–2021	152.7	50.3 ; 295.6			Upward
9 Marília	2011–2015	−20.9	−66 ; 14.5	6.1	−3.9 ; 20.8	Stationary/Stationary
	2015–2021	29.0	5.1 ; 137.8			Upward
10 Piracicaba	2011–2019	−7.6	−42.9 ; 2	9.0	0.4 ; 17.3	Stationary/Upward
	2019–2021	111.2	23.8 ; 222.8			Upward
15 São José do Rio Preto	2011–2021	11.8	2.2 ; 25	11.8	2.2 ; 25	Upward/Upward
16 Sorocaba	2011–2019	−2	−25.1 ; 11.5	18.2	9.5 ; 29.2	Stationary/Upward
	2019–2021	149.9	48.7 ; 290.8			Upward

Source: DATASUS (2023) *APC = Annual Percent Change; ^†^CI = Confidence Interval; ^‡^AAPC = Average Annual Percent Change

RHD 7 presented an inflection point separating the segments between 2011-2019 and 2019-2021, with an annual percent change of -2.3 of stationary trend in the first segment and in the second with a high annual percent change of 68.7%, with an upward trend, being the RHD with the highest APC in this period. Similarly, RHD 16 had an inflection point in the same year, with stationary APC in the first segment and upward in the next segment. However, both RHD 7 and 16 presented an AAPC with an upward trend in the total period of the series, as shown in Table 2.

Table 2 also shows the analysis of DMMR trends: most RHDs (1, 3, 4, 6, 7, 9, 10, 11, 15, 16 and 17) presented a stationary trend of the series, different from RHD 13, which presented an inflection point separating the segments between 2011-2015 and 2015-2021. In the first segment, there was a 39.8% growth trend in the annual percent change (95% CI 10.5 – 343.6). In the next segment, the trend was downward -12.9% annual percent change (95% CI -53.5 - -1.8). Thus, over the total period of the series, the DMMR remained stationary, with an average annual percent change of 5.3% (95% CI -11.4 – 26.2).

In the final part of Table 2, it is also possible to verify the IMMR trend analysis. RHD 1 presented an inflection point between 2011-2018 and 2018-2021, with a stationary trend of 2.4% in the first period (95% CI -8.7 – 7.3) and a growth trend of 32.1% in the annual percent change for the second period (95% CI 16.6 – 58.8). RHDs 4, 7, 10 and 16 presented inflection points between the periods 2011-2019 and 2019-2021. In the first segment detected, these RHDs showed a stationary trend for the annual percent change of 4% (95% CI -30.3 – 70), -4.1% (95% CI -29.8 – 8.3), -7.6% (95% CI -42.9 – 2) and -2% (95% CI -25.1 – 11.5), respectively. In the second segment, the RHDs showed an upward trend with an annual percent change of 50.6% (95% CI 4.4 – 107.7), 152.7% (95% CI 50.3; 295.6), 111.2% (95% CI 23.8 – 222.8) and 149.9% (95% CI 48.7 – 290.8).

RHD 9 presented an inflection point between the periods 2011-2015 and 2015-2021, with a stationary trend in the first period and an upward trend in the second period, with an annual percent change of -20.9% (95% CI -66 – 14.5) and 29.0% (95% CI 5.1 – 137.8). RHD 15 was the only one that did not present an inflection point, maintaining the upward trend throughout the series with an annual percent change of 11.8% (95% CI 2.2 – 25), as shown in Table 2.

## Discussion

This study analyzed the time-series distribution of MMR in the state of São Paulo and its RHDs between 2011 and 2021, enabling us to know the situation of maternal mortality before and during the COVID-19 pandemic.

Maternal mortality rates showed a significant and sudden increase during the pandemic. In the state of São Paulo, MMR had been showing a slight reduction between 2017 and 2019, but this trend was reversed in 2020 with the beginning of the pandemic. Another relevant aspect found in this study was the change in the profile of the causes of maternal deaths. In the period before the pandemic (2011-2019), deaths from direct causes were more common. However, during the pandemic years, there was an inversion, with a greater predominance of indirect causes. High maternal mortality rates represent not only a tragedy for families and society, but also a serious public health issue. Moreover, they are a reflection of the level of social development and a violation of women’s rights to health^(20,25)^.

Regarding the maternal mortality trend in the state, it was observed that the MMR time series remained stationary until 2019, followed by a trend of 25.6% growth in the annual percent change between 2019 and 2021. When fragmenting the series between DMMR and IMMR, it was observed that DMMR presented a distinct behavior from IMMR, exhibiting a growth trend of 7.2% for the annual percent change between 2011 and 2017, followed by a stationary trend between 2017 and 2021. On the other hand, IMMR remained stationary between 2011 and 2019, but showed an annual growth trend of 70.5% between 2019 and 2021, which may reflect the deaths reported as a result of COVID-19.

The slight reduction in cases of maternal mortality observed in the pre-pandemic period seems to reflect the actions adopted by Brazil and the state of São Paulo to strengthen health surveillance and health care for pregnant women. These initiatives include humanization policies at the national and state levels, the implementation of the Stork Network and the strategic training of members of the maternal, infant and fetal mortality committees in the state of São Paulo. However, with the advent of the COVID-19 pandemic, maintaining these rates became an unmet goal. The aggravation of socioeconomic inequalities, the shortage of hospital beds, the failures in the health care system and the difficulties in accessing services, in addition to the worsening in the quality of care provided, were determining factors for the significant increase in maternal deaths during this period^(26-27)^.

The trend analyses conducted in the RHDs showed that most areas followed the same pattern observed for the state, showing an upward trend for TMMR. Regarding the DMMR analyses, most areas exhibited a stationary behavior, with the exception of RHD 13 Ribeirão Preto, which, in the 2015-2021 period, showed a downward trend, with a 12.9% reduction in the annual percent change. On the other hand, IMMR showed a growth trend in the RHDs in which it was possible to establish an analysis model. Among them, the RHDs 7 Campinas, 16 Sorocaba and 10 Piracicaba presented the highest annual percent changes, with 152.7%, 149.9% and 111.2%, respectively.

During the COVID-19 pandemic, maternal mortality increased considerably in the state of São Paulo compared to the pre-pandemic period analyzed. These results are consistent with the national and international literature, especially for 2021, since the increase in maternal mortality during the pandemic was observed with greater impact in developing countries^(21,27)^.

This may reflect both the women’s difficulty in accessing health care services — since these services had their supply concentrated on meeting the demand of the health emergency — and the worsening of clinical conditions that SARS-CoV-2 infection causes in the health of pregnant and puerperal women^(28)^. Studies indicate that pregnancy does not increase the risk of women contracting COVID-19; however, it can aggravate the clinical outcome of the disease^(29)^. Pregnant and puerperal women with COVID-19, when compared to non-pregnant women, presented a higher risk of unfavorable outcomes, such as death, higher probability of developing severe acute respiratory syndrome (SARS) and need for admission to intensive care units^(20-21,30-34)^.

Brazilian studies found alarming results, indicating that the COVID-19 mortality rate among pregnant and puerperal women was almost three times higher than in the general population^(25)^. During the first wave of the pandemic, Brazil was responsible for 77.5% of all deaths of pregnant and puerperal women recorded in the medical literature in this period^(35)^. These data raise crucial questions about access to appropriate treatment and care, highlighting significant gaps in the health care system and medical care that contributed to these fatal outcomes. Corroborating these findings, other studies show that 5.9% of pregnant and puerperal women who died due to COVID-19 were not even hospitalized; 39.7% did not have access to ICU beds; 42.6% did not receive mechanical ventilation; and 25.5% did not have any ventilatory support^(36-37)^.

As shown above, the observed increase in maternal mortality was concentrated in low- and middle-income countries, which makes the issue even more concerning, considering that these countries have a significantly higher global maternal mortality burden. The reduced access to health care, the redistribution of teams working in maternity hospitals and the reduction of qualified personnel can compound the other conditions already mentioned, contributing to the increase in these adverse outcomes^(21)^.

An aspect noted in this study was the increase in maternal mortality in 2021. This increase may be related to the relaxation of social distancing measures, the spread of more contagious variants of the virus and increased mobility of the population; these are factors that contributed to a greater spread of the disease and consequent overload of the health care system^(38)^. Additional studies corroborate this finding, indicating a significant growth in maternal deaths in different states of Brazil, with an excess of 70% in deaths, especially in the North region, where rates were higher in the first half of 2021^(6,39-41)^.

Another point worth pointing out is the report in the literature on the increase in maternal mortality in the pandemic period among women with pregnancies of usual risk, when compared to the pre-pandemic period. Notably, the pandemic may have contributed to this increase in deaths of women with pregnancies of usual risk, probably due to disorder in the health care system, which usually already operates close to or above capacity^(41)^.

It is important to highlight that the severity of the effects caused by the COVID-19 pandemic and its high lethality may have been aggravated by the deficient planning and inconsistency in crisis management, especially with regard to the adoption of health regulations, the dissemination of information to the population, and the implementation of public health strategies to combat the disease^(42)^. In addition, there was a significant delay in the search for partnerships and in the development of research aimed at vaccines against SARS-CoV-2, which began only in early 2021, in a slow and disorganized manner. The inclusion of pregnant and puerperal women as priority groups for vaccination only occurred in July 2021, which may explain the higher concentration of deaths in the first half of that year. These management delays contrasted with the rapid spread of the virus and the emergence of new variants, possibly contributing to the increase in maternal mortality^(40,43-44)^.

The present study used secondary data; the chosen method entails certain limitations inherent to the study, such as the impossibility of establishing the exposure status of individuals, not necessarily meaning that the same association occurs individually, leading to the possibility of ecological bias.

Despite the limitations of this study, it contributes with new data on the researched issue, highlighting some relevant aspects. Notably, it is the first study to examine maternal mortality trends during the COVID-19 pandemic, considering the administrative divisions of RHDs in the state of São Paulo. This enables identifying the areas most affected by the increase in maternal deaths, allowing a critical analysis of the measures adopted in each location and comparisons between the results. Furthermore, the high coverage of SINASC and SIM records should be noted, as it reflects the quality and comprehensiveness of data for the population under analysis.

## Conclusion

The study findings show that the COVID-19 pandemic severely influenced the increase in maternal mortality rates in the state of São Paulo, especially deaths due to indirect obstetric causes. The year of 2021 had the highest rates recorded in the time series, representing an increase in MMR compared to previous years.

The health care system and its professionals undeniably need to develop and prioritize initiatives to reverse the situation found in this study. These initiatives should include maternal mortality prevention strategies, ensuring access to quality prenatal and obstetric care, in addition to a network design that embraces women with humanizing, person-centered care from pregnancy to puerperium.

Furthermore, it is essential to strengthen the work of maternal mortality committees by enabling them to expand their analyses and recommendations beyond maternal deaths, with monitoring strategies for future health emergencies and research on maternal *near-miss*. Reducing social, demographic, racial, gender and educational inequalities can also lead to better obstetric outcomes.

Finally, the evidence presented in this study is expected to contribute to the organization of strategies geared toward reducing maternal mortality by directing initiatives to priority areas of the state of São Paulo.

## Data Availability

All data generated or analysed during this study are available at https://doi.org/10.11606/T.22.2024.tde-12082024-130232.
