# Supplementary material for: Analysis of the influence of the COVID-19 pandemic on maternal mortality trends, 2011–2021
Source: Rev Lat Am Enfermagem. 2025 Nov 3;33:e4668. doi: 10.1590/1518-8345.7559.4668 (PMC12587756; doi:10.1590/1518-8345.7559.4668)
Supplement: Translation

# Translation: Análise da influência da pandemia de COVID-19 na tendência da mortalidade materna, 2011-2021*

**DOI:** 10.1590/1518-8345.7559.4669

**Authors:** Mariana Vitor Peppe, Fernanda Bruzadelli Paulino da Costa, Livia Sanches Pedrilio, Caio Antonio de Campos Prado, Ana Cyntia Paulin Baraldi, Juliana Stefanello

**Affiliations:** 1 Universidade de São Paulo, Escola de Enfermagem de Ribeirão Preto, Centro Colaborador da OPAS/OMS para o Desenvolvimento da Pesquisa em Enfermagem, Ribeirão Preto, SP, Brasil; 2 Bolsista da Coordenação de Aperfeiçoamento de Pessoal de Nível Superior (CAPES), Brasil; 3 Sociedade Beneficente Israelita Brasileira Albert Einstein, Escritório de Excelência, São Paulo, SP, Brasil; 4 Universidade de São Paulo, Faculdade de Medicina de Ribeirão Preto, Ribeirão Preto, SP, Brasil; 5 Fundação de Apoio ao Ensino, Pesquisa e Assistência, Centro de Referência da Saúde da Mulher-Mater, Ribeirão Preto, SP, Brasil; 6 Universidade de São Paulo, Hospital das Clínicas da Faculdade de Medicina de Ribeirão Preto, Ribeirão Preto, SP, Brasil; 7 Organização Pan-Americana da Saúde, Bireme, Brasília, DF, Brasil

**Keywords:** Morte Materna, COVID-19, Saúde Materna, Obstetrícia, Métodos Epidemiológicos, Sistema de Vigilância em Saúde

## Abstract

analisar a tendência temporal da mortalidade materna antes e durante a pandemia de COVID-19.

estudo ecológico de séries temporais, realizado no estado de São Paulo e em seus dezessete Departamentos Regionais de Saúde. Foram coletados dados secundários disponibilizados pelo Ministério da Saúde. Variáveis do estudo: razão de mortalidade materna total, direta e indireta. A análise foi realizada pelo modelo de regressão segmentada.

a tendência da razão de mortalidade materna foi estacionária entre 2011-2019, seguida de crescimento de 25,6% entre 2019-2021. A morte por causa direta teve uma tendência de crescimento de 7,2% no período 2011-2017 e uma tendência estacionária em 2017-2021. A razão de mortalidade por causa indireta foi estacionária entre 2011-2019, seguida de crescimento de 70,5% no período de 2019-2021. As regionais da Grande São Paulo, de Araçatuba, Bauru, Campinas, Presidente Prudente, São João da Boa Vista, São José do Rio Preto e Sorocaba apresentaram tendência de crescimento da morte materna entre 2011 e 2021.

a pandemia de COVID-19 influenciou severamente no aumento da morte materna, especialmente por causas indiretas, sendo 2021 o pior ano da série histórica. Evidenciou-se resultados importantes para auxiliar na organização de estratégias de redução da morte materna, direcionando-as para as regiões prioritárias.

## Destaques

**(1)** Contribuição para organização de estratégias para redução da morte materna. **(2)** Resultados importantes para a enfermagem planejar e implementar ações regionais. **(3)** Direcionamento das ações prioritárias para regiões com piores taxas de mortalidade. **(4)** A pandemia de COVID-19 impactou severamente o aumento da morte materna.

## Introdução

No início de 2020, a Organização Mundial da Saúde (OMS) anunciou a detecção de um grupo de casos de pneumonia na cidade de Wuhan, capital da província de Hubei, na China. Esses casos estavam associados a um novo coronavírus, até então desconhecido em humanos^(1)^. A doença rapidamente se espalhou por diversos países, tornando-se uma significativa causa de infecções respiratórias, pneumonia e mortes. Em março do mesmo ano, a OMS declarou a pandemia de COVID-19^(2-3)^, mantendo o estado de emergência em saúde pública até março de 2023^(4)^.

A pandemia de COVID-19 resultou em um aumento significativo no número de mortes por diferentes causas. De acordo com estimativas da OMS, houve aproximadamente 14,8 milhões de óbitos em excesso. O Brasil ficou em quinto lugar entre os países com o maior número absoluto de mortes além do esperado e ocupou a vigésima posição no *ranking* dos países com a maior proporção de óbitos excedentes em relação ao total esperado^(5)^. Esse cenário impactou diretamente na sobrecarga e no acesso aos sistemas de saúde, resultando na redução da disponibilidade de profissionais qualificados e recursos de saúde^(6)^. No começo da pandemia, especialistas estavam focados em determinar se a gestação poderia representar um fator de risco para complicações graves da COVID-19. Essa preocupação se baseava em evidências de desfechos semelhantes observados em infecções causadas por outros vírus respiratórios, como o H1N1^(7)^. Com a evolução da pandemia e das pesquisas, essa teoria se confirmou e evidenciou uma alta significativa nas taxas de mortalidade materna^(8-9)^. O aumento observado no Brasil provavelmente reflete problemas estruturais crônicos que afetam o atendimento à saúde da mulher em diferentes níveis de assistência. Entre esses desafios, destacam-se a dificuldade de acesso aos serviços de saúde e a baixa qualidade do atendimento pré-natal^(10)^.

A jornada das mulheres e dos profissionais de saúde brasileiros na área da obstetrícia tem sido longa na busca por um atendimento mais seguro ao parto e nascimento. Nas últimas três décadas, houve um fortalecimento das políticas nacionais voltadas à saúde materno-infantil, com foco na ampliação do acesso ao pré-natal e ao planejamento reprodutivo, visando melhorar a qualidade do cuidado obstétrico e reduzir a mortalidade materna. Dentre essas iniciativas, destacam-se a criação dos comitês de mortalidade materna e dos sistemas de vigilância de óbitos maternos, o Programa de Humanização do Pré-Natal e Nascimento, a Política Nacional de Atenção Integral à Saúde da Mulher, a Rede Cegonha^(11)^ e, mais recentemente, a Rede Alyne^(12)^. A implementação dessas medidas contribuiu para uma redução significativa da Razão de Mortalidade Materna (RMM) no Brasil, que passou de 72,4 para 57,9 óbitos maternos por 100 mil nascidos vivos entre 2009 e 2019^(13)^.

A redução das taxas de mortalidade materna é uma prioridade em nível global e integra a Agenda 2030 dos Objetivos de Desenvolvimento Sustentável (ODS) da Organização das Nações Unidas. A meta estabelecida busca diminuir a mortalidade materna mundial para menos de 70 óbitos a cada 100 mil nascidos vivos, servindo como referência para a formulação de políticas nacionais e iniciativas de cooperação internacional. Em 2015, o Brasil reafirmou seu compromisso com essa causa e ajustou a meta nacional, visando reduzir a Razão de Mortalidade Materna para, no máximo, 30 óbitos por 100 mil nascidos vivos^(14-15)^. Percebe-se que, nos anos anteriores à pandemia, houve uma estabilização da RMM em níveis que variaram de 62 mortes por 100 mil nascidos vivos em 2015 a 57,9 mortes por 100 mil nascidos vivos em 2019 com pequenas oscilações desde 2011^(16)^. Esses dados acendem um alerta sobre a possibilidade de o Brasil não cumprir a meta autoestabelecida em relação aos ODS^(13)^.

A morte materna é definida como o óbito de mulheres durante a gestação, abortamento, parto ou até 42 dias após o término da gestação, independentemente de sua duração. A causa está associada a condições relacionadas ou agravadas pela gravidez ou pelo seu manejo, excluindo-se fatores acidentais ou incidentais^(17-18)^. Diversos fatores influenciam a mortalidade materna, incluindo o acesso aos serviços de saúde, a qualidade da assistência prestada no pré-natal, durante o trabalho de parto, o parto, o puerpério e em emergências obstétricas. Além disso, fatores clínicos, socioeconômicos e a incidência de doenças também desempenham um papel significativo nesse cenário^(19-20)^.

Uma emergência de saúde pública pode agravar o cenário existente, elevando o risco de complicações na gestação, sobrecarregando o sistema de saúde e limitando o acesso aos cuidados maternos e perinatais^(20-21)^. Nesse contexto, a pandemia de COVID-19 demandou recursos assistenciais, humanos, institucionais, sociais, econômicos e políticos, competindo com outras necessidades do sistema de saúde. Como resultado, a mortalidade materna correu um risco ainda maior de ser negligenciada, especialmente por estar fortemente relacionada às desigualdades sociais.

Considerando as consequências da pandemia de COVID-19 na saúde materna, identificamos a necessidade de realizar este estudo para esclarecer a influência da pandemia na saúde das mulheres durante o ciclo gravídico-puerperal no estado de São Paulo. Essa necessidade, somada à carência de estudos regionais que analisem a mortalidade materna no contexto da pandemia por COVID-19, justifica a realização desse estudo. O objetivo do estudo foi analisar a tendência temporal da mortalidade materna antes e durante a pandemia de COVID-19.

## Método

### Delineamento do estudo

Para a realização deste estudo, adotamos uma abordagem epidemiológica com um estudo do tipo ecológico longitudinal analítico, por meio de séries temporais.

### Local e período do estudo

O estudo teve como unidade de análise os dezessete Departamentos Regionais de Saúde (DRS) do estado de São Paulo, cuja área territorial é de 248.219,481 km², abrigando 645 municípios e com uma população de 44.411.238 pessoas. Esse estado foi escolhido pois é o mais populoso do Brasil, tem um Índice de Desenvolvimento Humano (IDH) de 0,806, considerado muito alto^(22)^, e conta com uma rede administrativa de saúde organizada, contemplando um desenho assistencial satisfatório no contexto da linha de cuidado à saúde da gestante e puérpera. O período estudado abrangeu os dados dos anos anteriores à pandemia (2011 a 2019) e durante a pandemia (2020 a 2021). Para cada ano, foi considerado o intervalo de 1 de janeiro a 31 de dezembro.

### População

Foram coletadas informações agrupadas de mulheres gestantes, com faixa etária entre 10 e 49 anos, que evoluíram para o parto e que tiveram seus filhos nascidos vivos (NV), independente da via de parto, notificadas no Sistema de Informação Sobre Nascidos Vivos - SINASC. Também foram coletados dados de mulheres, com a mesma faixa etária, que estiveram gestantes e evoluíram para o óbito materno em decorrência de complicações da gestação, parto e pós-parto, tendo sido registrados no Sistema de Informação Sobre Mortalidade - SIM.

### Variáveis do estudo

As variáveis agregadas foram caracterizadas como: razão de mortalidade materna total (RMMT = número total de mortes maternas/número total de NV x 100.000), direta (RMMD = número total de mortes maternas de causa direta/número total de NV x 100.000) e indireta (RMMI = número total de mortes maternas de causa indireta/número total de NV x 100.000). Para todas as seleções realizadas no SINASC e SIM, foram consideradas as seguintes informações: município de residência e faixa etária de 10 a 49 anos.

A caracterização da morte materna foi realizada a partir dos diagnósticos estabelecidos na 10ª Revisão da Classificação Internacional de Doenças (CID-10), codificadas no capítulo XV – Complicações da gravidez, parto e puerpério, sendo classificados em morte obstétrica direta e indireta^(18)^. Também foram considerados os diagnósticos do CID-10 relacionados à gestação e ao puerpério incluídos em outros capítulos.

### Coleta dos dados

Os dados foram obtidos a partir do Sistema de Informação sobre Nascidos Vivos e do Sistema de Informação sobre Mortalidade, disponibilizados pelo Departamento de Informática do Sistema Único de Saúde (DATASUS), do Ministério da Saúde, por meio da plataforma TABNET. No SINASC foram coletadas informações agrupadas de mulheres que estiveram gestantes, que evoluíram para o parto e que tiveram seus filhos NV, independente da via de parto. No SIM, a coleta se deu a partir dos dados de mulheres que estiveram gestantes e que por decorrência de complicações da gestação, parto e pós-parto evoluíram para o óbito.

A coleta dos dados ocorreu entre os meses de setembro e novembro de 2023, sendo realizada pela própria pesquisadora. Durante a coleta, os dados brutos provenientes das bases de dados foram organizados e categorizados em planilha do *Microsoft Office Excel* ^®^ e, posteriormente, importados para as análises.

### Análise dos dados

Para análise dos dados das séries temporais, foram adotados modelos de regressão segmentados (*Joinpoint Regression*), construídos para identificar pontos de mudanças estatisticamente relevantes da RMM ao longo do tempo. Foram construídos modelos considerando as variáveis dependentes (razões de morte materna) e independentes (ano e DRS). Duas análises foram realizadas, a primeira considerando apenas os dados totais do estado de São Paulo, tendo como variáveis dependentes as razões de mortalidade materna (RMMT, RMMD e RMMI) e como variável independente os anos selecionados. Uma segunda análise foi realizada considerando a distribuição territorial das razões de mortalidade materna, mantendo essas razões como variável dependente e os DRS e anos como variáveis independentes.

A regressão segmentada é uma técnica de modelagem estatística que procura explicar a relação entre duas variáveis por meio de retas de regressão. Os pontos que unem essas retas são chamados de pontos inflexão^(23)^. Esse modelo assume uma tendência linear entre os pontos de inflexão e permite o ajuste de um modelo de regressão ponderado. Essa técnica estima a variação percentual anual (APC) e as variação percentual anual média (AAPC)^(24)^.

Foram considerados intervalos de confiança de 95% (IC 95%) e um nível de significância de 5%. O modelo foi ajustado para que o número de pontos de inflexão pudesse variar de zero a dois, ou seja, até três segmentos. Os testes de significância para escolha do melhor modelo foram baseados no método de permutação de Monte Carlo, e a implementação dos modelos foi realizada no programa *Joinpoint Regression Program* versão 4.3.1.0. A configuração padrão do próprio programa (método *Grid Search*) seleciona automaticamente o número de pontos necessários para ajustar cada segmento^(24)^.

### Aspectos éticos

O estudo foi aprovado pelo Comitê de Ética em Pesquisa sob o Parecer 4.649.272, de abril de 2021. Os dados utilizados são agregados e não permitem a identificação dos envolvidos nos resultados apresentados, dispensando assim a aplicação do Termo de Consentimento Livre e Esclarecido.

## Resultados

Foram registrados 3.339 óbitos maternos no estado de São Paulo, em mulheres com idade fértil entre 10 e 49 anos, entre 2011 e 2021. Considerando a RMM, notou-se uma pequena variação entre 2011 e 2016 (36,65 a 50,70 óbitos/100 mil NV), uma alta em 2017 (56,72 óbitos/100 mil NV), com uma queda de 7 óbitos/100 mil NV entre 2018 e 2019. Novo aumento aconteceu em 2020, com acréscimo de quase 12 óbitos/100 mil NV, chegando ao seu maior valor da série em 2021, com 87,02 óbitos/100 mil NV, conforme a Tabela 1.

**Tabela 1-:** Frequência relativa e razões de mortalidade materna por 100 mil nascidos vivos entre os anos. Estado de São Paulo, SP, Brasil, 2011-2021

	Anos												
			**2011**	**2012**	**2013**	**2014**	**2015**	**2016**	**2017**	**2018**	**2019**	**2020**	**2021**
Números absolutos													
Nascidos vivos*		n	610.150	616.545	610.836	625.590	633.935	652.732	611.735	606.065	583.081	552.210	525.134
Total óbito materno		n	248	226	240	263	310	331	347	303	282	332	457
Óbito materno direto*		n	152	125	146	150	191	199	212	189	158	154	143
		(%)	61,3	55,3	60,8	57,0	61,6	60,1	61,1	62,4	56,0	46,4	31,3
Óbito materno indireto*		n	89	97	90	105	113	126	119	104	113	158	301
		(%)	35,9	42,9	37,5	39,9	36,4	38,1	34,3	34,3	40,1	47,6	65,9
Óbito materno não especificado*		n	7	4	4	8	6	6	16	10	11	20	13
		(%)	2,8	1,8	1,7	3,0	1,9	1,7	4,6	3,3	3,9	6,0	2,8
													
RMM ^†^ total			40,6	36,6	39,3	42,0	48,9	50,7	56,7	50,0	48,4	60,1	87,0
RMM ^†^ direta			24,9	20,3	23,9	24,0	30,1	30,5	34,6	31,2	27,1	27,9	27,2
RMM ^†^ indireta			14,6	15,7	14,7	16,8	17,8	19,3	19,4	17,1	19,4	28,6	57,3
RMM ^†^ não especificada			1,1	0,6	0,6	1,3	0,94	0,9	2,6	1,6	1,9	3,6	2,5

*Fonte: DATASUS (2023); ^†^RMM = Razão de Mortalidade Materna

A Figura 1 demonstra a análise da série temporal. Os resultados observados revelam mudanças estatisticamente relevantes no que diz respeito à tendência das razões de mortalidade materna total, direta e indireta. A RMMT apresentou um ponto de inflexão, dividindo a série temporal em dois segmentos, 2011 a 2019 e 2019 a 2021. O primeiro segmento evidenciou um comportamento estacionário de 3,9% na variação percentual anual (IC 95% -12,4 – 25,5). O segundo segmento apontou uma tendência crescente da RMMT, tendo um aumento estatisticamente significativo de 25,6% na variação percentual anual (IC 95% 4,4 – 43,1), totalizando um crescimento na variação percentual anual média de 7,9%, com significância estatística (IC 95% 3,9 – 11,4) entre o período de 2011 e 2021.

Na Figura 2, observa-se a representação referente à RMMD, na qual foi detectado um ponto de inflexão, separando o primeiro segmento, correspondente aos anos de 2011 a 2017, que apresenta uma tendência de crescimento com uma variação percentual anual de 7,2% (IC 95% 3,9 – 22,8). No segundo segmento, de 2017 a 2021, embora o aspecto visual da curva seja descendente, estatisticamente a tendência foi estacionária, apresentando um percentual de 5,4% na RMMD (IC 95% -20,3 – 0,4). A variação percentual anual média para todo o período permaneceu estacionária em 2,0% (IC 95%: -1,3 – 5,6).

**Figura 1-:** Modelo de regressão *joinpoint* para a razão de mortalidade materna total, no estado de São Paulo

*Indica que a variação percentual anual (APC) é estatisticamente diferente de zero com alfa = 0,05; T Estatístico e valor-p não são disponíveis no método de *Empirical Quantile*; modelo final selecionado: 1 ponto de inflexão

**Figura 2-:** Modelo de regressão *joinpoint* para a razão de mortalidade materna por causa direta, no estado de São Paulo

*Indica que a variação percentual anual (APC) é estatisticamente diferente de zero com alfa = 0,05; T Estatístico e valor-p não são disponíveis no método de *Empirical Quantile*; modelo final selecionado: 1 ponto de inflexão

Conforme apresentado na Figura 3, dois segmentos foram identificados para a RMMI a partir de um ponto de inflexão. O primeiro, abrangendo os anos de 2011 a 2019, apresentou um comportamento estacionário na variação percentual anual, com um valor de 2,9% (IC 95% -0,5 – 5,7). O segundo segmento, corresponde ao período de 2019 a 2021, revelou uma tendência de crescimento na variação percentual anual de 70,5% (IC 95% 51,6 – 87,2). A variação percentual anual média, por sua vez, manteve-se em tendência de crescimento, registrando 13,8% (IC95% 11,8% - 16,1).

**Figura 3-:** Modelo de regressão *joinpoint* para a razão de mortalidade materna por causa indireta, no estado de São Paulo

*Indica que a variação percentual anual (APC) é estatisticamente diferente de zero com alfa = 0,05; T Estatístico e valor-p não são disponíveis no método de *Empirical Quantile*; Modelo final selecionado: 1 ponto de inflexão

Uma outra análise de regressão segmentada foi realizada a partir da distribuição da RMM entre os DRS. Alguns DRS não apresentaram óbito materno em determinados anos da série, impossibilitando, portanto, a obtenção de um modelo individualizado. Na análise da distribuição da RMMT nos DRS, observou-se que, no DRS 1, houve uma tendência de crescimento anual de 5,7% (IC95% 3,7 - 8). No DRS 2, a tendência de crescimento foi de 13,4% (IC95% 5,4 - 24,8). Os DRS 6, 11, 14 e 15 também apresentaram uma tendência de crescimento: 9,5% (IC95% 0,8 - 21,5), 20,01% (IC95% 8,29 – 39,12), 18,8% (IC95% 7,2 - 38,1) e 8% (IC95% 2,2 - 15,6), respectivamente, com ausência de *joinpoints*. Apesar dos DRS 3, 4, 9, 13, 17 apresentarem um valor positivo de APC e AAPC, a tendência foi considerada estacionária por não haver significância estatística, conforme exposto na Tabela 2.

**Tabela 2-:** Tendência da série temporal da razão de mortalidade materna total e por causas diretas e indiretas, utilizando a regressão segmentada, nos departamentos regionais de saúde. Estado de São Paulo, SP, Brasil, 2011-2021

Razão de morte materna total						
Departamentos Regionais de Saúde	**Períodos Segmentados**	**APC** *	**IC** ^†^ **95%**	AAPC ^‡^ (2011-2021)	**IC** ^†^ **95%**	Tendência APC*/AAPC ^‡^ APC *
1 Grande São Paulo	2011-2021	5,7	3,7 ; 8	5,7	3,7 ; 8	Crescente/Crescente
2 Araçatuba	2011-2021	13,4	5,4 ; 24,8	13,4	5,4 ; 24,8	Crescente/Crescente
3 Araraquara	2011-2021	2	-10,7 ; 5,9	2	-10,7 ; 15,9	Estacionária/Estacionária
4 Baixada Santista	2011-2021	7,4	-1,7 ; 18,2	7,4	-1,7 ; 18,2	Estacionária/Estacionária
6 Bauru	2011-2021	9,5	0,8 ; 21,5	9,5	0,8 ; 21,5	Crescente/Crescente
7 Campinas	2011-2019	- 2,3	-14,4 ; 3,6	8,9	3,4 ; 14,3	Estacionária/Crescente
	2019-2021	68,7	20,6 ; 18,5			Crescente
9 Marília	2011-2021	6,6	-0,2 ; 15,2	6,6	-0,2 ; 15,2	Estacionária/Estacionária
10 Piracicaba	2011-2013	-36,8	-63,2 ; 6,4	0,6	-7,3 ; 14,5	Estacionária/Estacionária
	2013-2021	13,1	-25 ; 96,3			Estacionária
11 Presidente Prudente	2011-2021	20,0	8,3 ; 9,1	20,0	8,3 ; 9,1	Crescente/Crescente
13 Ribeirão Preto	2011-2021	9,7	-1,3 ; 25,3	9,7	-1,3 ; 25,3	Estacionária/Estacionária
14 São João da Boa Vista	2011-2021	18,8	7,2 ; 38,1	18,8	7,2 ; 38,1	Crescente/Crescente
15 São José do Rio Preto	2011-2021	8,0	2,2 ; 15,6	8,0	2,2 ; 15,6	Crescente/Crescente
16 Sorocaba	2011-2019	1,8	-39,7 ; 0,5	12,5	3,2 ; 22,8	Estacionária/Crescente
	2019-2021	68,2	10,3 ; 48,9			Crescente
17 Taubaté	2011-2021	7,6	-4,9 ; 23,3	7,6	-4,9 ; 23,3	Estacionária/Estacionária
Razão de morte materna por causas diretas						
1 Grande São Paulo	2011-2017	6,0	2,1 ; 26,2	1,1	-2,6 ; 4,9	Crescente/Estacionária
	2017-2021	-5,9	-24,1 – 0,5			Estacionária
3 Araraquara	2011-2021	-2,9	-19 ; 14,3	-2,9	-19 ; 14,3	Estacionária/ Estacionária
4 Baixada Santista	2011-2021	3,2	-4 ; 11,5	3,2	-4 ; 11,5	Estacionária/ Estacionária
6 Bauru	2011-2019	12,1	-1,9 ; 184,4	-8	-26,9 ; 23,1	Estacionária/ Estacionária
	2019-2021	-58,4	- 88,4 ; 5			Estacionária
7 Campinas	2011-2021	1	-5,7 ; 8	1	-5,7 ; 8	Estacionária/ Estacionária
9 Marília	2011-2017	18,9	-4,4 ; 195,5	3,8	-12,2 ; 25,9	Estacionária/ Estacionária
	2017-2021	-15,3	-70,8 ; 16,1			Estacionária
10 Piracicaba	2011-2021	1,3	-8 ; 11,7	1,3	-8 ; 11,7	Estacionária/ Estacionária
11 Presidente Prudente	2011-2021	7,2	-10,5 ; 31,3	7,2	-10,5 ; 31,3	Estacionária/ Estacionária
13 Ribeirão Preto	2011-2015	39,8	10,5 ; 343,6	5,3	-11,4 ; 26,2	Crescente/Estacionária
	2015-2021	-12,9	-53,5 ; -1,8			Decrescente
15 São José do Rio Preto	2011-2021	2,7	-2,7 ; 8,8	2,7	-2,7 ; 8,8	Estacionária/ Estacionária
16 Sorocaba	2011-2021	7,8	-2,6 ; 22	7,8	-2,6 ; 22	Estacionária/ Estacionária
17 Taubaté	2011-2021	-0,1	-13,2 ; 15,4	-0,1	-13,2 ; 15,4	Estacionária/ Estacionária
Razão de morte materna por causas indiretas						
1 Grande São Paulo	2011-2018	2,4	-8,7 ; 7,3	10,5	7,2 ; 13,6	Estacionária/Crescente
	2018-2021	32,1	16,6 ; 58,8			Crescente
4 Baixada Santista	2011-2019	4	-30,3 ; 70	12	3,3 ; 22,5	Estacionária/Crescente
	2019-2021	50,6	4,4 ; 107,7			Crescente
7 Campinas	2011-2019	-4,1	-29,8 ; 8,3	16,4	7,7 ; 27,1	Estacionária/Crescente
	2019-2021	152,7	50,3 ; 295,6			Crescente
9 Marília	2011-2015	-20,9	-66 ; 14,5	6,1	-3,9 ; 20,8	Estacionária/Estacionária
	2015-2021	29,0	5,1 ; 137,8			Crescente
10 Piracicaba	2011-2019	-7,6	-42,9 ; 2	9,0	0,4 ; 17,3	Estacionária/Crescente
	2019-2021	111,2	23,8 ; 222,8			Crescente
15 São José do Rio Preto	2011-2021	11,8	2,2 ; 25	11,8	2,2 ; 25	Crescente/Crescente
16 Sorocaba	2011-2019	-2	-25,1 ; 11,5	18,2	9,5 ; 29,2	Estacionária/Crescente
	2019-2021	149,9	48,7 ; 290,8			Crescente

Fonte: DATASUS (2023) *APC = *Annual Percent Change*; ^†^IC = Intervalo de confiança; ^‡^AAPC = *Average Annual Percent Change*

O DRS 7 apresentou um ponto de inflexão separando os segmentos entre 2011-2019 e 2019-2021, com uma variação percentual anual -2,3 de tendência estacionária no primeiro segmento e no segundo com uma alta variação percentual anual de 68,7%, com tendência crescente, sendo o DRS com maior APC nesse período. Da mesma maneira, o DRS 16 teve um ponto de inflexão no mesmo ano, com APC estacionário no primeiro segmento e crescente no segmento seguinte. Entretanto, tanto o DRS 7 quanto o 16 apresentaram um AAPC com tendência crescente no período total da série, como visto na Tabela 2.

A Tabela 2 ainda revela a análise das tendências da RMMD: a maioria dos DRS (1, 3, 4, 6, 7, 9, 10, 11, 15, 16 e 17) apresentou uma tendência estacionária da série, diferente do DRS 13, que apresentou um ponto de inflexão separando os segmentos entre 2011-2015 e 2015-2021. No primeiro segmento, houve tendência de crescimento de 39,8% na variação percentual anual (IC95% 10,5 – 343,6), já no segmento seguinte, a tendência foi decrescente -12,9% de variação percentual anual (IC95% -53,5 - -1,8). Assim, ao longo do período total da série, a RMMD manteve-se estacionária, com variação percentual anual média de 5,3% (IC95% -11,4 – 26,2).

Na parte final da Tabela 2, também é possível verificar a análise da tendência da RMMI. O DRS 1 apresentou um ponto de inflexão entre 2011-2018 e 2018-2021, com tendência estacionária de 2,4% no primeiro período (IC 95% -8,7 – 7,3) e tendência de crescimento de 32,1% na variação percentual anual, para o segundo período (IC95% 16,6 – 58,8). Os DRS 4, 7, 10 e 16 apresentaram pontos de inflexão entre os períodos 2011-2019 e 2019-2021. No primeiro segmento detectado, esses DRS apresentaram tendência estacionária para a variação percentual anual de 4% (IC95% -30,3 – 70), -4,1% (IC95%-29,8 – 8,3), -7,6% (IC95% -42,9 – 2) e -2% (IC95% -25,1 – 11,5), respectivamente. No segundo segmento, os DRS apresentaram tendência de crescimento com variação percentual anual de 50,6% (IC95% 4,4 – 107,7), 152,7% (IC95% 50,3; 295,6), 111,2% (IC95% 23,8 – 222,8) e 149,9% (IC95% 48,7 – 290,8).

O DRS 9 apresentou um ponto de inflexão entre os períodos 2011-2015 e 2015-2021, com tendência estacionária no primeiro período e crescente no segundo período, com variação percentual anual de -20,9% (IC95% -66 – 14,5) e 29,0% (IC95% 5,1 – 137,8). O DRS 15 foi o único que não apresentou ponto de inflexão, mantendo a tendência crescente no decorrer da série com variação percentual anual de 11,8% (IC95% 2,2 – 25), conforme demonstrado na Tabela 2.

## Discussão

Este estudo analisou a distribuição temporal da RMM no estado de São Paulo e seus DRS entre os anos de 2011 e 2021, permitindo conhecer a realidade da mortalidade materna antes e durante a pandemia de COVID-19.

As taxas de mortalidade materna apresentaram um aumento significativo e repentino durante a pandemia. No estado de São Paulo, a RMM vinha apresentando uma leve redução entre 2017 e 2019, mas essa tendência foi revertida em 2020 com o início da pandemia. Outro aspecto relevante identificado neste estudo foi a mudança no perfil das causas dos óbitos maternos. No período anterior à pandemia (2011-2019), as mortes por causas diretas eram mais comuns. No entanto, durante os anos pandêmicos, ocorreu uma inversão, com maior predominância de causas indiretas. Altas taxas de mortalidade materna representam não apenas uma tragédia para as famílias e a sociedade, mas também um grave problema de saúde pública. Além disso, são um reflexo do nível de desenvolvimento social e uma violação dos direitos das mulheres à saúde^(20,25)^.

Em relação à tendência da mortalidade materna no estado, observou-se que a série temporal da RMM permaneceu estacionária até 2019, seguida por uma tendência de crescimento de 25,6% na variação percentual anual entre 2019 e 2021. Ao fragmentar a série entre RMMD e RMMI, notou-se que a RMMD apresentou um comportamento distinto da RMMI, exibindo uma tendência de crescimento de 7,2% na variação percentual anual entre 2011 e 2017, seguida por uma tendência estacionária entre 2017 a 2021. Por outro lado, a RMMI manteve-se estacionária entre 2011 e 2019, mas apresentou uma tendência de crescimento anual de 70,5% entre 2019 e 2021, podendo ser reflexo das mortes notificadas em decorrência da COVID-19.

A leve redução nos casos de mortalidade materna observada no período pré-pandêmico parece refletir as ações adotadas pelo Brasil e pelo estado de São Paulo para fortalecer a vigilância e a assistência à saúde da gestante. Entre essas iniciativas, destacam-se as políticas de humanização em nível nacional e estadual, a implementação da Rede Cegonha e a capacitação estratégica dos membros dos comitês de mortalidade materna, infantil e fetal do estado de São Paulo. No entanto, com a chegada da pandemia de COVID-19, a manutenção dessas taxas tornou-se um objetivo não alcançado. O agravamento das desigualdades socioeconômicas, a escassez de leitos, as falhas no sistema de saúde e as dificuldades de acesso aos serviços, somadas à piora na qualidade dos cuidados prestados, foram fatores determinantes para o expressivo aumento das mortes maternas durante esse período^(26-27)^.

As análises de tendência conduzidas nos DRS mostraram que a maior parte das regiões seguiu o mesmo padrão observado no estado, apresentando tendência de crescimento para a RMMT. Em relação às análises da RMMD, a maioria das regiões exibiu um comportamento estacionário, com exceção do DRS 13 Ribeirão Preto, que, no período de 2015-2021, apresentou uma tendência decrescente, com uma redução de 12,9% na variação percentual anual. Por outro lado, a RMMI demonstrou uma tendência de crescimento nos DRS em que foi possível estabelecer um modelo de análise. Entre eles, os DRS 7 Campinas, 16 Sorocaba e 10 Piracicaba apresentaram as maiores variações percentuais anuais, registrando 152,7%, 149,9% e 111,2%, respectivamente.

Durante a pandemia de COVID-19, a mortalidade materna aumentou consideravelmente no estado de São Paulo em comparação com o período pré-pandêmico analisado. Esses resultados estão em consonância com a literatura nacional e internacional, especialmente no ano de 2021, uma vez que o aumento da mortalidade materna durante a pandemia foi observado com maior impacto nos países em desenvolvimento^(21,27)^.

Isso pode refletir tanto a dificuldade das mulheres em acessar os serviços de saúde, já que a oferta desses serviços foi concentrada no atendimento à demanda da urgência sanitária, quanto o agravamento das condições clínicas que a infecção por SARS-CoV-2 causa na saúde de gestantes e puérperas^(28)^. Pesquisas indicam que a gestação não aumenta o risco da mulher contrair COVID-19, entretanto pode agravar o desfecho clínico da doença^(29)^. Gestantes e puérperas com COVID-19, quando comparadas a mulheres não grávidas, apresentaram um risco maior de desfechos desfavoráveis, como o óbito, maior probabilidade de desenvolver síndrome respiratória aguda grave (SRAG) e necessidade de admissão em unidades de terapia intensiva^(20-21,30-34)^.

Estudos brasileiros revelaram resultados alarmantes, indicando que a taxa de letalidade da COVID-19 entre gestantes e puérperas foi quase três vezes maior do que na população geral^(25)^. Durante a primeira onda da pandemia, o Brasil foi responsável por 77,5% de todas as mortes de gestantes e puérperas registradas na literatura médica nesse período^(35)^. Esses dados levantam questões cruciais sobre o acesso ao tratamento e cuidados adequados, evidenciando falhas significativas no sistema de saúde e na assistência médica, que contribuíram para esses desfechos fatais. Corroborando esses achados, outros estudos mostram que 5,9% das gestantes e puérperas que faleceram devido à COVID-19 nem sequer foram internadas; 39,7% não tiveram acesso a leitos de UTI; 42,6% não receberam ventilação mecânica e 25,5% não contaram com qualquer suporte ventilatório^(36-37)^.

Como exposto anteriormente, o aumento observado da mortalidade materna foi concentrado nos países de baixa e média renda, o que torna a questão ainda mais preocupante, considerando que esses países apresentam uma carga global de mortalidade materna significativamente maior. A redução no acesso aos cuidados de saúde, a redistribuição das equipes atuantes em maternidades e a diminuição de pessoal qualificado podem somar-se às outras condições já mencionadas, contribuindo para o aumento desses desfechos adversos^(21)^.

Um aspecto destacado neste estudo foi o aumento da mortalidade materna no ano de 2021. Esse crescimento pode estar relacionado ao relaxamento das medidas de distanciamento social, à disseminação de variantes mais contagiosas do vírus e ao aumento da mobilidade da população, fatores que contribuíram para uma maior disseminação da doença e consequente sobrecarga do sistema de saúde^(38)^. Estudos adicionais corroboram essa constatação, indicando um crescimento expressivo nas mortes maternas em diferentes estados do Brasil, com um excesso de 70% nos óbitos, especialmente na região Norte, onde os índices foram mais elevados no primeiro semestre de 2021^(6,39-41)^.

Outro ponto que merece atenção é o relato na literatura sobre o aumento da mortalidade materna no período pandêmico entre mulheres com gestações de risco habitual, quando comparado ao período pré-pandêmico. Destaca-se que a pandemia pode ter contribuído para esse aumento nas mortes de mulheres com gestações de risco habitual, provavelmente devido a desarranjos no sistema de saúde, que, habitualmente, já opera próximo ou acima de sua capacidade^(41)^.

É importante destacar que a gravidade dos efeitos ocasionados pela pandemia de COVID-19 e sua alta letalidade podem ter sido agravadas pela falta de planejamento e pela incoerência na gestão da crise, especialmente no que se refere à adoção de regulamentações sanitárias, à disseminação de informações à população e à implementação de estratégias de saúde pública para o enfrentamento da doença^(42)^. Além disso, houve um atraso significativo na busca por parcerias e no desenvolvimento de pesquisas voltadas para as vacinas contra o SARS-CoV-2, cujo início ocorreu apenas no começo de 2021, de maneira lenta e desorganizada. A inclusão de gestantes e puérperas como grupos prioritários para a vacinação só aconteceu em julho de 2021, o que pode justificar a maior concentração de óbitos no primeiro semestre desse ano. Esses atrasos na gestão contrastaram com a rápida disseminação do vírus e o surgimento de novas variantes, possivelmente contribuindo para o aumento da mortalidade materna^(40,43-44)^.

O presente estudo foi realizado com dados secundários; o próprio método escolhido acarreta certas limitações inerentes ao estudo, como a impossibilidade de estabelecer o status de exposição dos indivíduos, não significando, obrigatoriamente, que a mesma associação ocorra de maneira individual, gerando a possibilidade do viés ecológico.

Apesar das limitações deste estudo, ele contribui com novos dados sobre o tema investigado, destacando alguns aspectos relevantes. Notavelmente, trata-se da primeira pesquisa a examinar as tendências da mortalidade materna durante a pandemia de COVID-19, levando em consideração as divisões administrativas dos DRS no estado de São Paulo. Isso possibilita a identificação das regiões mais afetadas pelo aumento dos óbitos maternos, permitindo uma análise crítica das medidas adotadas em cada localidade e a realização de comparações entre os resultados. Além disso, a alta cobertura dos registros do SINASC e do SIM deve ser ressaltada, pois reflete a qualidade e abrangência dos dados da população analisada.

## Conclusão

Os resultados deste estudo evidenciam que a pandemia de COVID-19 influenciou severamente o aumento das taxas de mortalidade materna no estado de São Paulo, principalmente nas mortes decorrentes de causas obstétricas indiretas. O ano de 2021 apresentou as maiores taxas registradas na série histórica, representando um aumento na RMM em comparação aos anos anteriores.

É inegável a necessidade de que o sistema de saúde e os profissionais nele atuantes elaborem e priorizem ações para reverter o cenário evidenciado neste estudo. Essas ações devem incluir estratégias para a prevenção de óbitos maternos, garantindo acesso ao pré-natal e à assistência obstétrica de qualidade, além de um desenho de rede que acolha a mulher desde a pré-concepção até o puerpério.

Adicionalmente, é fundamental fortalecer a atuação dos comitês de mortalidade materna, permitindo que expandam suas análises e recomendações para além das mortes maternas, com estratégias de monitoramento para futuras emergências sanitárias e investigação do *near miss* materno. A redução de desigualdades sociais, demográficas, raciais, de gênero e educacionais também pode refletir em melhores desfechos obstétricos.

Por fim, espera-se que as evidências apresentadas neste estudo contribuam para a organização de estratégias voltadas à redução da mortalidade materna, direcionando esforços para as regiões prioritárias do estado de São Paulo.

## Data Availability

Todos os dados gerados ou analisados durante este estudo estão disponíveis em https://doi.org/10.11606/T.22.2024.tde-12082024-130232.

# Translation: Análisis de la influencia de la pandemia de COVID-19 en la tendencia de la mortalidad materna, 2011-2021*

**DOI:** 10.1590/1518-8345.7559.4667

**Authors:** Mariana Vitor Peppe, Fernanda Bruzadelli Paulino da Costa, Livia Sanches Pedrilio, Caio Antonio de Campos Prado, Ana Cyntia Paulin Baraldi, Juliana Stefanello

**Affiliations:** 1 Universidade de São Paulo, Escola de Enfermagem de Ribeirão Preto, Centro Colaborador de la OPS/OMS para el Desarrollo de la Investigación en Enfermería, Ribeirão Preto, SP, Brasil; 2 Becaria de la Coordenação de Aperfeiçoamento de Pessoal de Nível Superior (CAPES), Brasil; 3 Sociedade Beneficente Israelita Brasileira Albert Einstein, Escritório de Excelência, São Paulo, SP, Brasil; 4 Universidade de São Paulo, Faculdade de Medicina de Ribeirão Preto, Ribeirão Preto, SP, Brasil; 5 Fundação de Apoio ao Ensino, Pesquisa e Assistência, Centro de Referência da Saúde da Mulher-Mater, Ribeirão Preto, SP, Brasil; 6 Universidade de São Paulo, Hospital das Clínicas da Faculdade de Medicina de Ribeirão Preto, Ribeirão Preto, SP, Brasil; 7 Organização Pan-Americana da Saúde, Bireme, Brasília, DF, Brasil

**Keywords:** Muerte Materna, COVID-19, Salud Materna, Obstetricia, Métodos Epidemiológicos, Sistema de Vigilancia Sanitaria

## Abstract

analizar la tendencia temporal de la mortalidad materna antes y durante la pandemia de COVID-19.

estudio ecológico de series temporales, realizado en el estado de São Paulo y en sus diecisiete Departamentos Regionales de Salud. Se recopilaron datos secundarios proporcionados por el Ministerio de Salud. Variables del estudio: razón de mortalidad materna total, directa e indirecta. El análisis se realizó por modelo de regresión segmentada.

la tendencia de la razón de mortalidad materna fue estacionaria entre 2011-2019, seguida de un incremento del 25,6% entre 2019-2021. La muerte por causa directa tuvo una tendencia de incremento del 7,2% en el período 2011-2017 y una tendencia estacionaria en 2017-2021. La tasa de mortalidad por causa indirecta se mantuvo estacionaria entre 2011-2019, seguida de un incremento del 70,5% en el período 2019-2021. Las regiones de Grande São Paulo, Araçatuba, Bauru, Campinas, Presidente Prudente, São João da Boa Vista, São José do Rio Preto y Sorocaba mostraron una tendencia de crecimiento de muerte materna entre el 2011 y el 2021.

la pandemia de COVID-19 influyó severamente en el incremento de las muertes maternas, especialmente por causas indirectas, y el 2021 fue el peor año de la serie histórica. Se destacaron resultados importantes para ayudar a organizar estrategias para reducir las muertes maternas, orientándolas hacia regiones prioritarias.

## Destacados

**(1)** Contribución a la organización de estrategias para reducir las muertes maternas. **(2)** Resultados importantes para que la enfermería pueda planificar e implementar acciones regionales. **(3)** Dirigir acciones prioritarias a las regiones con peores tasas de mortalidad. **(4)** La pandemia de COVID-19 ha tenido un impacto severo en el incremento de las muertes maternas.

## Introducción

A principios del 2020, la Organización Mundial de la Salud (OMS) anunció la detección de un grupo de casos de neumonía en la ciudad de Wuhan, capital de la provincia de Hubei, China. Estos casos se asociaron con un nuevo coronavirus, hasta entonces desconocido en humanos^(1)^. La enfermedad se propagó rápidamente a varios países, convirtiéndose en una significativa causa de infecciones respiratorias, neumonía y muertes. En marzo del mismo año, la OMS declaró la pandemia de COVID-19^(2-3)^, manteniendo el estado de emergencia de salud pública hasta marzo del 2023^(4)^.

La pandemia de COVID-19 produjo un aumento significativo en el número de muertes por diferentes causas. Según estimaciones de la OMS, hubo aproximadamente 14,8 millones de muertes adicionales. Brasil se situó en el quinto lugar entre los países con mayor número absoluto de muertes más allá de lo esperado y ocupó el vigésimo lugar en el ranking de países con mayor proporción de muertes excedentes con relación al total esperado^(5)^. Este escenario impactó directamente en la sobrecarga y en el acceso a los sistemas de salud, y produjo una reducción en la disponibilidad de profesionales cualificados y recursos de salud^(6)^. Al principio de la pandemia, los expertos se centraron en determinar si el embarazo podía suponer un factor de riesgo para complicaciones graves a causa de la COVID-19. Esta preocupación se basó en evidencias de desenlaces similares observados en infecciones causadas por otros virus respiratorios, como el H1N1^(7)^. Con la evolución de la pandemia y de los estudios, esta teoría se confirmó y mostró un aumento significativo en las tasas de mortalidad materna^(8-9)^. El aumento observado en Brasil probablemente refleja problemas estructurales crónicos que afectan la atención de la salud de la mujer en diferentes niveles de asistencia. Entre estos desafíos, destacan la dificultad de acceso a los servicios de salud y la baja calidad de la atención prenatal^(10)^.

El recorrido de las mujeres brasileñas y de los profesionales de la salud en el área de la obstetricia ha sido largo en la búsqueda de una atención más segura durante el parto y el nacimiento. En las últimas tres décadas, se fortalecieron las políticas nacionales orientadas a la salud materna e infantil, con un enfoque en la ampliación del acceso a la atención prenatal y la planificación reproductiva, para mejorar la calidad de la atención obstétrica y reducir la mortalidad materna. Entre estas iniciativas, destacan la creación de comités de mortalidad materna y sistemas de vigilancia de muertes maternas, el Programa de Humanización del Prenatal y del Nacimiento, la Política Nacional de Atención Integral a la Salud de la Mujer, la Rede Cegonha^(11)^ y, más recientemente, la Rede Alyne^(12)^. La implementación de estas medidas contribuyó a una reducción significativa de la Razón de Mortalidad Materna (RMM) en Brasil, que pasó de 72,4 a 57,9 muertes maternas por cada 100.000 nacidos vivos entre el 2009 y el 2019^(13)^.

Reducir las tasas de mortalidad materna es una prioridad mundial y forma parte de la Agenda 2030 de los Objetivos de Desarrollo Sostenible (ODS) de las Naciones Unidas. La meta establecida busca reducir la mortalidad materna mundial a menos de 70 muertes por cada 100.000 nacidos vivos, y sirve como referencia para la formulación de políticas nacionales e iniciativas de cooperación internacional. En el 2015, Brasil reafirmó su compromiso con esta causa y ajustó la meta nacional, con el objetivo de reducir la Razón de Mortalidad Materna a un máximo de 30 muertes por cada 100.000 nacidos vivos^(14-15)^. Se puede observar que, en los años previos a la pandemia, la RMM se estabilizó en niveles que oscilaron entre 62 muertes por 100.000 nacidos vivos en el 2015 hasta 57,9 muertes por 100.000 nacidos vivos en el 2019, con pequeñas fluctuaciones desde el 2011^(16)^. Estos datos encienden una alerta por la posibilidad de que Brasil no alcance la meta autoestablecida con relación a los ODS^(13)^.

La muerte materna se define como la muerte de la mujer durante el embarazo, el aborto, el parto o hasta 42 días después del término del embarazo, independientemente de su duración. La causa está asociada a condiciones relacionadas o agravadas por el embarazo o por su manejo, excluyendo factores accidentales o incidentales^(17-18)^. Diversos factores influyen en la mortalidad materna, entre ellos el acceso a los servicios de salud, la calidad de la asistencia prestada en el período prenatal, durante el trabajo de parto, el parto, el puerperio y en emergencias obstétricas. Además, los factores clínicos, socioeconómicos y la incidencia de enfermedades también desempeñan un papel significativo en este escenario^(19-20).^.

Una emergencia de salud pública puede empeorar el escenario existente y aumentar el riesgo de complicaciones durante el embarazo, sobrecargando el sistema de salud y limitando el acceso a los cuidados maternos y perinatales^(20-21)^. En este contexto, la pandemia de COVID-19 demandó recursos asistenciales, humanos, institucionales, sociales, económicos y políticos, compitiendo con otras necesidades del sistema de salud. En consecuencia, la mortalidad materna sufrió un riesgo aún mayor de ser descuidada, especialmente porque está fuertemente relacionada con las desigualdades sociales.

Considerando las consecuencias de la pandemia de COVID-19 en la salud materna, identificamos la necesidad de realizar este estudio para aclarar la influencia de la pandemia en la salud de las mujeres durante el ciclo embarazo-puerperal en el estado de São Paulo. Esta necesidad, sumada a la falta de estudios regionales que analicen la mortalidad materna en el contexto de la pandemia de COVID-19, justifica la realización de este estudio. El objetivo del estudio fue analizar la tendencia temporal de la mortalidad materna antes y durante la pandemia de COVID-19.

## Método

### Diseño del estudio

Para realizar este estudio, adoptamos un enfoque epidemiológico con un estudio de carácter ecológico longitudinal analítico, por medio de series temporales.

### Lugar y período de estudio

El estudio tuvo como unidad de análisis los diecisiete Departamentos Regionales de Salud (DRS) del estado de São Paulo, cuya área territorial es de 248.219,481 km², con 645 municipios y una población de 44.411.238 de personas. Se eligió este estado por ser el más poblado de Brasil y por tener un Índice de Desarrollo Humano (IDH) de 0,806, considerado muy alto^(22)^, y por contar con una red administrativa de salud organizada, que incluye un diseño asistencial satisfactorio en el contexto de la línea de cuidado a la salud de la mujer embarazada y puérpera. El período estudiado abarcó datos de los años previos a la pandemia (2011 a 2019) y durante la pandemia (2020 a 2021). Para cada año, se consideró el intervalo del 1 de enero al 31 de diciembre.

### Población

Se recopiló información agrupada de mujeres embarazadas, con edades comprendidas entre 10 y 49 años, que progresaron al parto y cuyos hijos nacieron vivos (NV), independientemente de la modalidad del parto, reportados en el Sistema de Información de Nacidos Vivos (SINASC). También se recopilaron datos de mujeres, del mismo rango de edad, que estuvieron embarazadas y fallecieron debido a complicaciones del embarazo, parto y posparto, y cuyo fallecimiento se registró en el Sistema de Información sobre Mortalidad (SIM).

### Variables del estudio

Las variables agregadas se caracterizaron como: razón de mortalidad materna total (RMMT = número total de muertes maternas/número total de NV x 100.000), directa (RMMD = número total de muertes maternas por causas directas/número total de NV x 100.000) e indirecta (RMMI = número total de muertes maternas por causas indirectas/número total de NV x 100.000). Para todas las selecciones realizadas en el SINASC y el SIM se consideró la siguiente información: municipio de residencia y rango de edad de 10 a 49 años.

La caracterización de la muerte materna se realizó a partir de los diagnósticos establecidos en la 10.ª Revisión de la Clasificación Internacional de Enfermedades (CIE-10), codificados en el capítulo XV: Complicaciones del embarazo, parto y puerperio, clasificándose en muerte obstétrica directa e indirecta^(18)^. También se consideraron los diagnósticos de la CIE-10 relacionados con el embarazo y el puerperio incluidos en otros capítulos.

### Recopilación de datos

Los datos se obtuvieron del Sistema de Información de Nacidos Vivos y del Sistema de Información de Mortalidad, proporcionados por el Departamento de Informática del Sistema Único de Salud (DATASUS), del Ministerio de Salud, mediante la plataforma TABNET. En el SINASC se recopiló información agrupada de mujeres que estuvieron embarazadas, que progresaron al parto y que tuvieron a sus hijos NV, independientemente de la vía de parto. En el SIM, la recopilación se realizó a partir de los datos de mujeres que estuvieron embarazadas y que fallecieron como consecuencia de complicaciones durante el embarazo, el parto y el posparto.

La recopilación de datos tuvo lugar entre septiembre y noviembre del 2023, y fue realizada por la propia investigadora. Durante la recopilación, los datos brutos provenientes de las bases de datos se organizaron y se categorizaron en una hoja de cálculo de *Microsoft Office Excel* ^®^ y, posteriormente, se importaron para los análisis.

### Análisis de datos

Para el análisis de datos de series temporales se adoptaron modelos de regresión segmentados (*Joinpoint Regression*), construidos para identificar puntos de cambios estadísticamente relevantes de la RMM a lo largo del tiempo. Se construyeron modelos considerando las variables dependientes (tasas de mortalidad materna) e independientes (año y DRS). Se realizaron dos análisis, el primero considerando solo los datos totales del estado de São Paulo, con las razones de mortalidad materna (RMMT, RMMD y RMMI) como variables dependientes y los años seleccionados como variable independiente. Se realizó un segundo análisis considerando la distribución territorial de las razones de mortalidad materna, manteniendo dichas razones como variable dependiente y la DRS y los años como variable independiente.

La regresión segmentada es una técnica de modelado estadístico que busca explicar la relación entre dos variables mediante rectas de regresión. Los puntos que unen estas rectas se llaman puntos de inflexión^(23)^. Este modelo asume una tendencia lineal entre puntos de inflexión y permite el ajuste de un modelo de regresión ponderado. Esta técnica estima la variación porcentual anual (APC) y la variación porcentual anual media (AAPC)^(24)^.

Se consideraron intervalos de confianza del 95% (IC95%) y un nivel de significancia del 5%. El modelo fue ajustado para que el número de puntos de inflexión pudiera variar de cero a dos, es decir, hasta tres segmentos. Las pruebas de significancia para elegir el mejor modelo se basaron en el método de permutación de Monte Carlo, y la implementación de los modelos se realizó en el programa *Joinpoint Regression Program* versión 4.3.1.0. La configuración estándar del propio programa (método *Grid Search*) selecciona automáticamente el número de puntos necesarios para ajustar cada segmento^(24)^.

### Aspectos éticos

El estudio fue aprobado por el Comité de Ética en Investigación bajo el Dictamen 4.649.272, de abril del 2021. Los datos utilizados son agregados y no permiten la identificación de los participantes en los resultados presentados, por lo que prescinde de la aplicación del Consentimiento Libre, Previo e Informado.

## Resultados

Se registraron 3.339 muertes maternas en el estado de São Paulo, en mujeres de 10 a 49 años, entre el 2011 y el 2021. Considerando la RMM, se observó una pequeña variación entre el 2011 y el 2016 (36,65 a 50,70 muertes/100.000 NV), un aumento en el 2017 (56,72 muertes/100.000 NV), con una reducción de 7 muertes/100.000 NV entre el 2018 y el 2019. Un nuevo incremento se produjo en el 2020, con un incremento de casi 12 muertes/100.000 NV, alcanzando su valor más alto de la serie en el 2021, con 87,02 muertes/100.000 NV, según la Tabla 1.

**Tabla 1-:** Frecuencia relativa y razones de mortalidad materna por 100.000 nacidos vivos entre años. Estado de São Paulo, SP, Brasil, 2011-2021

	Años												
			**2011**	**2012**	**2013**	**2014**	**2015**	**2016**	**2017**	**2018**	**2019**	**2020**	**2021**
Números absolutos													
Nacidos vivos*		n	610,150	616,545	610,836	625,590	633,935	652,732	611,735	606,065	583,081	552,210	525,134
Muertes maternas totales		n	248	226	240	263	310	331	347	303	282	332	457
Muertes maternas directas*		n	152	125	146	150	191	199	212	189	158	154	143
		(%)	61,3	55,3	60,8	57,0	61,6	60,1	61,1	62,4	56,0	46,4	31,3
Muertes maternas indirectas*		n	89	97	90	105	113	126	119	104	113	158	301
		(%)	35,9	42,9	37,5	39,9	36,4	38,1	34,3	34,3	40,1	47,6	65,9
Muertes maternas no especificadas*		n	7	4	4	8	6	6	16	10	11	20	13
		(%)	2,8	1,8	1,7	3,0	1,9	1,7	4,6	3,3	3,9	6,0	2,8
Total de RMM ^†^			40,6	36,6	39,3	42,0	48,9	50,7	56,7	50,0	48,4	60,1	87,0
RMM ^†^ directa			24,9	20,3	23,9	24,0	30,1	30,5	34,6	31,2	27,1	27,9	27,2
RMM ^†^ indirecta			14,6	15,7	14,7	16,8	17,8	19,3	19,4	17,1	19,4	28,6	57,3
RRM ^†^ no especificada			1,1	0,6	0,6	1,3	0,94	0,9	2,6	1,6	1,9	3,6	2,5

*Fuente: DATASUS (2023); ^†^RMM = Razón de Mortalidad Materna

La Figura 1 demuestra el análisis de la serie de temporal. Los resultados observados revelan cambios estadísticamente relevantes respecto a la tendencia de las razones de mortalidad materna total, directa e indirecta. El RMMT presentó un punto de inflexión, dividiendo la serie temporal en dos segmentos, 2011 a 2019 y 2019 a 2021. El primer segmento mostró un comportamiento estacionario del 3,9% en la variación porcentual anual (IC95% -12,4 – 25,5). El segundo segmento indicó una tendencia creciente de la RMMT, con un aumento estadísticamente significativo del 25,6% en la variación porcentual anual (IC95% 4,4 – 43,1), totalizando un crecimiento en la variación porcentual anual promedio del 7,9%, con significancia estadística (IC95% 3,9 – 11,4) entre el período del 2011 y el 2021.

En la Figura 2, se puede observar la representación referente a la RMMD, en la cual se detectó un punto de inflexión, separando el primer segmento, correspondiente a los años 2011 a 2017, el cual presenta una tendencia de crecimiento con una variación porcentual anual del 7,2% (IC95% 3,9 – 22,8). En el segundo segmento, del 2017 al 2021, si bien el aspecto visual de la curva es descendente, estadísticamente la tendencia fue estacionaria y presenta un porcentaje del 5,4% en la RMMD (IC95% -20,3 – 0,4). La variación porcentual anual media para todo el período permaneció estacionaria en un 2,0% (IC95% -1,3 – 5,6).

**Figura 1-:** Modelo de regresión *joinpoint* para la razón de mortalidad materna total, en el estado de São Paulo

*Indica que la variación porcentual anual (APC) es estadísticamente diferente de cero con alfa = 0,05; T estadístico y p-valor no disponibles para el método *Empirical Quantile*; modelo final seleccionado: 1 *joinpoint*

**Figura 2-:** Modelo de regresión *joinpoint* para la razón de mortalidad materna directa, en el estado de São Paulo

*Indica que la variación porcentual anual (APC) es estadísticamente diferente de cero con alfa = 0,05; T estadístico y p-valor no disponibles para el método *Empirical Quantile*; modelo final seleccionado: 1 *joinpoint*

Como se muestra en la Figura 3, se identificaron dos segmentos para la RMMI desde un punto de inflexión. El primero, que abarca los años del 2011 al 2019, mostró un comportamiento estacionario en la variación porcentual anual, con un valor del 2,9% (IC95% -0,5 – 5,7). El segundo segmento, correspondiente al período 2019-2021, reveló una tendencia de crecimiento en la variación porcentual anual de 70,5% (IC95% 51,6 – 87,2). La variación porcentual anual media, a su vez, se mantuvo en una tendencia ascendente, registrándose en un 13,8% (IC95% 11,8% - 16,1).

**Figura 3-:** Modelo de regresión *joinpoint* para la razón de mortalidad materna indirecta, en el estado de São Paulo

*Indica que la variación porcentual anual (APC) es estadísticamente diferente de cero con alfa = 0,05; T estadístico y p-valor no disponibles para el método *Empirical Quantile*; modelo final seleccionado: 1 *joinpoint*

Se realizó otro análisis de regresión segmentada basado en la distribución del RMM entre los DRS. Algunos DRS no presentaron muertes maternas en ciertos años de la serie, por lo que no fue posible obtener un modelo individualizado. En el análisis de la distribución de RMMT en los DRS, se observó que, en el DRS 1, hubo una tendencia de crecimiento anual del 5,7% (IC95% 3,7 – 8). En el DRS 2, la tendencia de crecimiento fue del 13,4% (IC95%: 5,4 - 24,8). Los DRS 6, 11, 14 y 15 también mostraron una tendencia de incremento: 9,5% (IC95%: 0,8-21,5), 20,01% (IC95%: 8,29-39,12), 18,8% (IC95%: 7,2-38,1) y 8% (IC95%: 2,2-15,6), respectivamente, con ausencia de *joinpoints*. Aunque los DRS 3, 4, 9, 13, 17 presentaron un valor positivo de APC y AAPC, la tendencia se consideró estacionaria al no existir significancia estadística, como se muestra en la Tabla 2.

**Tabla 2-:** Tendencia de la serie temporal de la razón de mortalidad materna total y por causas directas e indirectas, mediante regresión segmentada, en los departamentos regionales de salud. Estado de São Paulo, SP, Brasil, 2011-2021

Razón de mortalidad materna total						
Departamentos Regionales de Salud	**Períodos segmentados**	**APC** *	**IC** ^†^ **95%**	AAPC ^‡^ (2011-2021)	**IC** ^†^ **95%**	Tendencia APC*/AAPC ^‡^ APC *
1 Grande São Paulo	2011-2021	5,7	3,7 ; 8	5,7	3,7 ; 8	Ascendente/Ascendente
2 Araçatuba	2011-2021	13,4	5,4 ; 24,8	13.4	5,4 ; 24,8	Ascendente/Ascendente
3 Araraquara	2011-2021	2	-10,7 ; 5.9	2	-10,7 ; 15,9	Estacionaria/Estacionaria
4 Baixada Santista	2011-2021	7,4	-1,7 ; 18,2	7,4	-1,7 ; 18,2	Estacionaria/Estacionaria
6 Bauru	2011-2021	9,5	0,8 ; 21,5	9,5	0,8 ; 21,5	Ascendente/Ascendente
7 Campinas	2011-2019	- 2,3	-14,4 ; 3,6	8,9	3,4 ; 14,3	Estacionaria/Ascendente
	2019-2021	68,7	20,6 ; 18,5			Ascendente
9 Marília	2011-2021	6,6	-0,2 ; 15,2	6,6	-0,2 ; 15,2	Estacionaria/Estacionaria
10 Piracicaba	2011-2013	-36,8	-63,2 ; 6,4	0,6	-7,3 ; 14,5	Estacionaria/Estacionaria
	2013-2021	13,1	-25 ; 96,3			Estacionaria
11 Presidente Prudente	2011-2021	20,0	8,3 ; 9,1	20,0	8,3 ; 9,1	Ascendente/Ascendente
13 Ribeirão Preto	2011-2021	9,7	-1,3 ; 25,3	9,7	-1,3 ; 25,3	Estacionaria/Estacionaria
14 São João da Boa Vista	2011-2021	18,8	7,2 ; 38,1	18,8	7,2 ; 38,1	Ascendente/Ascendente
15 São José do Rio Preto	2011-2021	8,0	2,2 ; 15,6	8,0	2,2 ; 15,6	Ascendente/Ascendente
16 Sorocaba	2011-2019	1,8	-39,7 ; 0,5	12,5	3,2 ; 22,8	Estacionaria/Ascendente
	2019-2021	68,2	10,3 ; 48,9			Ascendente
17 Taubaté	2011-2021	7,6	-4,9 ; 23,3	7,6	-4,9 ; 23,3	Estacionaria/Estacionaria
Razón de mortalidad materna por causas directas						
1 Grande São Paulo	2011-2017	6,0	2,1 ; 26,2	1,1	-2,6 ; 4,9	Ascendente/Estacionaria
	2017-2021	-5,9	-24,1 – 0,5			Estacionaria
3 Araraquara	2011-2021	-2,9	-19 ; 14,3	-2,9	-19 ; 14,3	Estacionaria/Estacionaria
4 Baixada Santista	2011-2021	3,2	-4 ; 11,5	3,2	-4 ; 11,5	Estacionaria/Estacionaria
6 Bauru	2011-2019	12,1	-1,9 ; 184,4	-8	-26,9 ; 23,1	Estacionaria/Estacionaria
	2019-2021	-58,4	- 88,4 ; 5			Estacionaria
7 Campinas	2011-2021	1	-5,7 ; 8	1	-5,7 ; 8	Estacionaria/Estacionaria
9 Marília	2011-2017	18,9	-4,4 ; 195,5	3,8	-12,2 ; 25,9	Estacionaria/Estacionaria
	2017-2021	-15,3	-70,8 ; 16,1			Estacionaria
10 Piracicaba	2011-2021	1,3	-8 ; 11,7	1,3	-8 ; 11,7	Estacionaria/Estacionaria
11 Presidente Prudente	2011-2021	7,2	-10,5 ; 31,3	7,2	-10,5 ; 31,3	Estacionaria/Estacionaria
13 Ribeirão Preto	2011-2015	39,8	10,5 ; 343,6	5,3	-11,4 ; 26,2	Ascendente/Estacionaria
	2015-2021	-12,9	-53,5 ; -1,8			Descendente
15 São José do Rio Preto	2011-2021	2,7	-2,7 ; 8,8	2,7	-2,7 ; 8,8	Estacionaria/Estacionaria
16 Sorocaba	2011-2021	7,8	-2,6 ; 22	7,8	-2,6 ; 22	Estacionaria/Estacionaria
17 Taubaté	2011-2021	-0,1	-13,2 ; 15,4	-0,1	-13,2 ; 15,4	Estacionaria/Estacionaria
Razón de mortalidad materna por causas indirectas						
1 Grande São Paulo	2011-2018	2,4	-8,7 ; 7,3	10,5	7,2 ; 13,6	Estacionaria/Ascendente
	2018-2021	32,1	16,6 ; 58,8			Ascendente
4 Baixada Santista	2011-2019	4	-30,3 ; 70	12	3,3 ; 22,5	Estacionaria/Ascendente
	2019-2021	50,6	4,4 ; 107,7			Ascendente
7 Campinas	2011-2019	-4,1	-29,8 ; 8,3	16,4	7,7 ; 27,1	Estacionaria/Ascendente
	2019-2021	152,7	50,3 ; 295,6			Ascendente
9 Marília	2011-2015	-20,9	-66 ; 14,5	6,1	-3,9 ; 20,8	Estacionaria/Estacionaria
	2015-2021	29,0	5,1 ; 137,8			Ascendente
10 Piracicaba	2011-2019	-7,6	-42,9 ; 2	9,0	0,4 ; 17,3	Estacionaria/Ascendente
	2019-2021	111,2	23,8 ; 222,8			Ascendente
15 São José do Rio Preto	2011-2021	11,8	2,2 ; 25	11,8	2,2 ; 25	Ascendente/Ascendente
16 Sorocaba	2011-2019	-2	-25,1 ; 11,5	18,2	9,5 ; 29,2	Estacionaria/Ascendente
	2019-2021	149,9	48,7 ; 290,8			Ascendente

Fuente: DATASUS (2023) *APC = *Annual Percent Change*; ^†^IC = Intervalo de confianza; ^‡^AAPC = *Average Annual Percent Change*

El DRS 7 presentó un punto de inflexión separando los segmentos entre 2011-2019 y 2019-2021, con una variación porcentual anual de -2,3 con tendencia estacionaria en el primer segmento y en el segundo con una alta variación porcentual anual del 68,7%, con tendencia creciente, y fue el DRS con mayor APC en este período. De manera similar, el DRS 16 tuvo un punto de inflexión en el mismo año, con el APC estacionario en el primer segmento y ascendente en el segmento siguiente. Sin embargo, tanto el DRS 7 como el DRS 16 presentaron un AAPC con tendencia ascendente a lo largo de todo el período de la serie, como se observa en la Tabla 2.

La Tabla 2 también revela el análisis de las tendencias de la RMMD: la mayoría de los DRS (1, 3, 4, 6, 7, 9, 10, 11, 15, 16 y 17) presentaron una tendencia estacionaria en la serie, a diferencia del DRS 13, que presentó un punto de inflexión separando los segmentos entre 2011-2015 y 2015-2021. En el primer segmento, se observó una tendencia de incremento del 39,8% en la variación porcentual anual (IC95% 10,5 – 343,6). En el siguiente segmento, la tendencia fue decreciente -12,9% de variación porcentual anual (IC95% -53,5 –1,8). Así, a lo largo del período total de la serie, la RMMD se mantuvo estacionaria, con una variación porcentual anual media del 5,3% (IC95% -11,4 – 26,2).

En la parte final de la Tabla 2, también es posible comprobar el análisis de la tendencia RMMI. El DRS 1 mostró un punto de inflexión entre 2011-2018 y 2018-2021, con una tendencia estacionaria del 2,4% en el primer período (IC95% -8,7 – 7,3) y una tendencia de crecimiento del 32,1% en el cambio porcentual anual para el segundo período (IC95% 16,6 – 58,8). Los DRS 4, 7, 10 y 16 mostraron puntos de inflexión entre los períodos 2011-2019 y 2019-2021. En el primer segmento detectado, estos DRS mostraron una tendencia estacionaria para la variación porcentual anual del 4% (IC95% -30,3 – 70), -4,1% (IC95% -29,8 – 8,3), -7,6% (IC95% -42,9 – 2) y -2% (IC95% -25,1 – 11,5), respectivamente. En el segundo segmento, los DRS mostraron una tendencia de crecimiento con una variación porcentual anual del 50,6% (IC95% 4,4 – 107,7), 152,7% (IC95% 50,3; 295,6), 111,2% (IC95% 23,8 – 222,8) y 149,9% (IC95% 48,7 – 290,8).

El DRS 9 presentó un punto de inflexión entre los períodos 2011-2015 y 2015-2021, con una tendencia estacionaria en el primer período y ascendente en el segundo período, con una variación porcentual anual del -20,9% (IC95% -66 – 14,5) y 29,0% (IC95% 5,1 – 137,8). El DRS 15 fue el único que no presentó un punto de inflexión, manteniendo la tendencia ascendente a lo largo de la serie con una variación porcentual anual del 11,8% (IC95% 2,2 – 25), como se muestra en la Tabla 2.

## Discusión

Este estudio analizó la distribución temporal de la RMM en el estado de São Paulo y sus DRS entre los años 2011 y 2021, lo que permitió conocer la realidad de la mortalidad materna antes y durante la pandemia de COVID-19.

Las tasas de mortalidad materna presentaron un aumento significativo y repentino durante la pandemia. En el estado de São Paulo, la RMM venía presentando una ligera reducción entre el 2017 y el 2019, pero esa tendencia se revirtió en el 2020 con el inicio de la pandemia. Otro aspecto relevante identificado en este estudio fue el cambio en el perfil de las causas de muertes maternas. En el período previo a la pandemia (2011-2019), las muertes por causas directas eran más frecuentes. Sin embargo, durante los años de pandemia se produjo una reversión, con un mayor predominio de las causas indirectas. Las altas tasas de mortalidad materna no solo representan una tragedia para las familias y la sociedad, sino también un grave problema de salud pública. Además, son un reflejo del nivel de desarrollo social y una violación de los derechos de las mujeres a la salud^(20,25)^.

Con respecto a la tendencia de la mortalidad materna en el estado, se observó que la serie temporal de la RMM se mantuvo estacionaria hasta el 2019, seguida de una tendencia ascendente del 25,6% en la variación porcentual anual entre el 2019 y el 2021. Al fragmentar la serie entre RMMD y RMMI, se observó que la RMMD presentó un comportamiento diferente a la RMMI y mostró una tendencia de incremento del 7,2% en la variación porcentual anual entre el 2011 y el 2017, seguida de una tendencia estacionaria entre el 2017 y el 2021. Por otra parte, la RMMI se mantuvo estacionaria entre el 2011 y el 2019, pero mostró una tendencia de incremento anual del 70,5% entre el 2019 y el 2021, lo que puede ser un reflejo de las muertes reportadas como consecuencia de la COVID-19.

La ligera reducción de los casos de mortalidad materna observada en el período prepandémico parece reflejar las acciones adoptadas por Brasil y por el estado de São Paulo para fortalecer la vigilancia y la asistencia a la salud de la mujer embarazada. Entre estas iniciativas, destacan las políticas de humanización en el ámbito nacional y estatal, la implementación de la Rede Cegonha y la capacitación estratégica de miembros de los comités de mortalidad materna, infantil y fetal del estado de São Paulo. Sin embargo, con la llegada de la pandemia de COVID-19, mantener estas tasas se convirtió en un objetivo no alcanzado. El agravamiento de las desigualdades socioeconómicas, la escasez de camas, las fallas del sistema de salud y las dificultades de acceso a los servicios, sumados al empeoramiento en la calidad de los cuidados brindados fueron factores determinantes del aumento significativo de las muertes maternas durante este período^(26-27)^.

Los análisis de tendencia realizados en los DRS mostraron que la mayor parte de las regiones siguió el mismo estándar observado en el estado, lo que reveló una tendencia de incremento para la RMMT. En cuanto a los análisis de la RMMD, la mayoría de las regiones mostró un comportamiento estacionario, salvo el DRS 13 Ribeirão Preto, que, en el período de 2015-2021, presentó una tendencia descendente, con una reducción del 12,9% en su variación porcentual anual. Por otra parte, la RMMI mostró una tendencia de incremento en los DRS en la que fue posible establecer un modelo de análisis. Entre ellos, los DRS 7 Campinas, 16 Sorocaba y 10 Piracicaba presentaron las mayores variaciones porcentuales anuales, registrando 152,7%, 149,9% y 111,2%, respectivamente.

Durante la pandemia de COVID-19, la mortalidad materna aumentó considerablemente en el estado de São Paulo en comparación con el período prepandémico analizado. Estos resultados están en línea con la literatura nacional e internacional, especialmente en el 2021, ya que el aumento de la mortalidad materna durante la pandemia se observó con mayor impacto en los países en desarrollo^(21,27)^.

Esto puede reflejar tanto la dificultad que tienen las mujeres para acceder a los servicios de salud, ya que la oferta de dichos servicios se concentró en atender la demanda de la urgencia sanitaria, como el agravamiento de las condiciones clínicas que la infección por SARS-CoV-2 provocó en la salud de las mujeres embarazadas y puérperas^(28)^. Las investigaciones indican que el embarazo no aumenta el riesgo de que una mujer contraiga COVID-19, sin embargo puede empeorar el desenlace clínico de la enfermedad^(29)^. Las mujeres embarazadas y puérperas con COVID-19, en comparación con las mujeres no embarazadas, presentaron un mayor riesgo de desenlaces desfavorables, como muerte, una mayor probabilidad de desarrollar síndrome respiratorio agudo severo (SARS) y la necesidad de ingreso a unidades de cuidados intensivos^(20-21,30-34)^.

Estudios brasileños revelaron resultados alarmantes e indicaron que la tasa de mortalidad por COVID-19 entre mujeres embarazadas y puérperas fue casi tres veces mayor que en la población general^(25)^. Durante la primera ola de la pandemia, Brasil fue responsable del 77,5% de todas las muertes de mujeres embarazadas y puérperas registradas en la literatura médica durante ese período^(35)^. Estos datos plantean preguntas cruciales sobre el acceso al tratamiento y cuidados adecuados, y ponen de relieve importantes fallos en el sistema de salud y en la atención médica que contribuyeron a estos desenlaces fatales. Corroborando estos hallazgos, otros estudios muestran que el 5,9% de las mujeres embarazadas y puérperas que fallecieron a causa de COVID-19 ni siquiera fueron hospitalizadas; el 39,7% no tuvo acceso a camas de UCI; el 42,6% no recibió ventilación mecánica; y el 25,5% no tuvo ningún soporte ventilatorio^(36-37)^.

Como se mencionó anteriormente, el incremento observado de la mortalidad materna se concentró en los países de ingresos bajos y medios, lo que hace que el problema sea aún más preocupante, considerando que estos países tienen una carga mundial de mortalidad materna significativamente mayor. La reducción en el acceso a los cuidados de salud, la redistribución de los equipos que trabajan en las maternidades y la disminución del personal cualificado pueden sumarse a las otras condiciones ya mencionadas, lo que contribuye al aumento de estos desenlaces adversos^(21)^.

Un aspecto destacado en este estudio fue el incremento de la mortalidad materna en el 2021. Este incremento puede estar relacionado con la relajación de las medidas de distanciamiento social, la propagación de variantes más contagiosas del virus y la mayor movilidad de la población, factores que contribuyeron a una mayor propagación de la enfermedad y consecuente sobrecarga del sistema de salud^(38)^. Estudios adicionales corroboran este hallazgo e indican un aumento significativo de muertes maternas en diferentes estados de Brasil, con un exceso del 70% en las muertes, especialmente en la región Norte, donde las tasas fueron más altas en el primer semestre del 2021^(6,39-41)^.

Otro punto que merece atención es el reporte en la literatura sobre el aumento de la mortalidad materna durante el período pandémico entre mujeres con embarazos de riesgo habitual, en comparación con el período prepandémico. Cabe señalar que la pandemia puede haber contribuido a este aumento de muertes de mujeres con embarazos de riesgo habitual, probablemente debido a alteraciones en el sistema de salud, que, habitualmente ya opera cerca o por encima de su capacidad^(41)^.

Es importante destacar que la gravedad de los efectos causados por la pandemia de COVID-19 y su alta letalidad pueden haberse visto agravados por la falta de planificación y por la incoherencia en la gestión de la crisis, especialmente en lo que respecta a la adopción de regulaciones sanitarias, la difusión de información a la población y la implementación de estrategias de salud pública para el afrontamiento de la enfermedad^(42)^. Además, hubo un retraso importante en la búsqueda de alianzas y en el desarrollo de investigaciones centradas en vacunas contra el SARS-CoV-2, que recién comenzaron a principios del 2021, de manera lenta y desorganizada. La inclusión de mujeres embarazadas y puérperas como grupos prioritarios para la vacunación recién ocurrió en julio del 2021, lo que puede justificar la mayor concentración de muertes en el primer semestre de este año. Estos retrasos en la gestión contrastaron con la rápida propagación del virus y la aparición de nuevas variantes, lo que posiblemente contribuyó al aumento de la mortalidad materna^(40,43-44)^.

El presente estudio se realizó con datos secundarios; el propio método elegido conlleva ciertas limitaciones inherentes al estudio, como la imposibilidad de establecer el estatus de exposición de los individuos, lo que no significa, necesariamente, que la misma asociación se produzca de forma individual, generando la posibilidad de sesgo ecológico.

A pesar de las limitaciones de este estudio, aporta nuevos datos sobre el tema investigado, destacando algunos aspectos relevantes. Cabe destacar que este es el primer estudio que examina las tendencias de la mortalidad materna durante la pandemia de COVID-19, teniendo en cuenta las divisiones administrativas de los DRS en el estado de São Paulo. Esto permite identificar las regiones más afectadas por el aumento de las muertes maternas, lo que permite un análisis crítico de las medidas adoptadas en cada localidad y comparaciones entre los resultados. Además, cabe destacar la alta cobertura de los registros del SINASC y del SIM, ya que refleja la calidad y el alcance de los datos sobre la población analizada.

## Conclusión

Los resultados de este estudio muestran que la pandemia de COVID-19 influyó severamente en el aumento de las tasas de mortalidad materna en el estado de São Paulo, sobre todo en las muertes resultantes de causas obstétricas indirectas. El año 2021 presentó las tasas más altas registradas en la serie histórica, lo que representa un incremento en la RMM en comparación con los años anteriores.

Es innegable la necesidad de que el sistema de salud y los profesionales que en él trabajan desarrollen y prioricen acciones para revertir el escenario resaltado en este estudio. Estas acciones deben incluir estrategias para prevenir muertes maternas, garantizar el acceso a la atención prenatal y a la atención obstétrica de calidad, además de un diseño de red que apoye a las mujeres desde la preconcepción hasta el puerperio.

Asimismo, es fundamental fortalecer el trabajo de los comités de mortalidad materna, permitiendo que expandan sus análisis y recomendaciones más allá de las muertes maternas, con estrategias de monitoreo para futuras emergencias sanitarias e investigación del *near miss* materno. La reducción de las desigualdades sociales, demográficas, raciales, de género y educativas también puede resultar en mejores desenlaces obstétricos.

Finalmente, se espera que las evidencias presentadas en este estudio contribuyan a la organización de estrategias encaminadas a la reducción de la mortalidad materna, dirigiendo esfuerzos hacia las regiones prioritarias del estado de São Paulo.

## Data Availability

Todos los datos generados o analizados durante este estudio están disponibles en https://doi.org/10.11606/T.22.2024.tde-12082024-130232.
